# How the Plants for Joints multidisciplinary lifestyle intervention achieved its effects: a mixed methods process evaluation

**DOI:** 10.1186/s12889-024-18554-2

**Published:** 2024-04-13

**Authors:** Carlijn A. Wagenaar, Alie Toonstra, Wendy Walrabenstein, Dirkjan van Schaardenburg, Femke van Nassau

**Affiliations:** 1 Reade Center for Rheumtology and Rehabilitation, Amsterdam, The Netherlands; 2 Department of Clinical Immunology and Rheumatology, Amsterdam University Medical Centers, Universiteit van Amsterdam, Amsterdam, The Netherlands; 3 Amsterdam Rheumatology and Immunology Center, Amsterdam, The Netherlands; 4 Department of Public and Occupational Health, Amsterdam University Medical Centers, Vrije Universiteit Amsterdam, Amsterdam Public Health Research Institute, Amsterdam, The Netherlands

**Keywords:** Process evaluation, Lifestyle intervention, Rheumatoid arthritis, Osteoarthritis

## Abstract

**Background:**

Plants for Joints (PFJ) is a multidisciplinary intervention centered around a whole-food plant-based diet, physical activity, and sleep and stress management. The PFJ intervention successfully improved disease activity and symptoms in people with rheumatoid arthritis (RA) or osteoarthritis (OA), respectively, and metabolic health. To investigate how these effects were achieved a mixed methods process evaluation was conducted to understand the context, implementation, and mechanism of impact of the PFJ intervention. Also, the relationship between degree of implementation and lifestyle changes was explored.

**Methods:**

Quantitative and qualitative data were collected across the evaluation domains context (i.e. reach), implementation (i.e. recruitment and delivery), and mechanism of impact (i.e. responsiveness) of both the participants and coaches (incl. dietitians, sport coaches) according to the UK MRC guidelines for process evaluations. Data was collected from the participants via focus groups and questionnaires after the intervention, and interviews with coaches. Qualitative data were analyzed thematically, and quantitative data were assessed with descriptive statistics and linear regression analyses. Degree of implementation was quantified using a theory-driven implementation index score composed of different process evaluation constructs.

**Results:**

Of the 155 participants who participated in the PFJ intervention, 106 (68%) took part in the questionnaire and 34 (22%) attended a focus group. Participants felt the intervention was complete, coherent, and would recommend the intervention to others (mean score 9.2 (SD 1.4) out of 10). Participants felt heard and empowered to take control of their lifestyle and health outcomes. Components perceived as most useful were self-monitoring, social support, practical and theoretical information, and (individual) guidance by the multidisciplinary team. Participants perceived the intervention as feasible, and many indicated it effectively improved their health outcomes. In an explorative analysis there was no significant difference in healthy lifestyle changes across implementation index score groups.

**Conclusion:**

This process evaluation offers important insights into why the PFJ intervention works and how the intervention can be optimized for future implementation. Results indicating the intervention’s high satisfaction, feasibility, and perceived effectiveness, further support the use of plant-based lifestyle interventions as an additional treatment option for patients with RA, OA, or other chronic diseases.

**Trial registration:**

International Clinical Trial Registry Platform numbers: NL7800, NL7801, and NL7802, all registered 17-06-2019.

**Supplementary Information:**

The online version contains supplementary material available at 10.1186/s12889-024-18554-2.

## Background

Non-communicable diseases (NCDs) are the leading cause of death worldwide and encompass a wide range of conditions including cardiovascular disease, type 2 diabetes, but also rheumatoid arthritis (RA) and osteoarthritis (OA) [1]. Unhealthy lifestyle behaviors, such as poor diet, obesity, physical inactivity, disturbed sleep, and stress are drivers of low-grade chronic inflammation and thereby increase the risk of the development and progression of NCDs [2]. Consequently, interventions directed at modifying these risk factors and targeting chronic inflammation are needed for the prevention and management of NCDs [2–6]. As people with RA and OA also face a greater risk of developing other chronic diseases, including cardiovascular disease, lifestyle interventions play an additional role in the secondary prevention of comorbidities [2, 7, 8].

Previous studies show lifestyle interventions with plant-based diets significantly improve health outcomes in people with cardiovascular disease, type 2 diabetes, and obesity [9–11]. Recently, the ‘Plants for Joints’ (PFJ) multidisciplinary lifestyle intervention, including a whole food plant-based diet, physical activity, and sleep and stress management significantly improved disease activity in people with RA [12], and pain, stiffness, and physical function in people with OA compared to usual care [13]. Both RA and OA groups had improved metabolic outcomes, including significant reductions in weight, fat mass, HbA1c, and LDL-cholesterol [12, 13]. Given the effectiveness of the PFJ intervention on improving disease specific outcomes and (risk factors of) comorbidities, the intervention can potentially be used as an additional treatment option for people with RA, OA, and other NCDs.

Process evaluations explore the ways complex interventions, such as PFJ, are implemented, and can help explain why they work and how they can be optimized [14]. The input gathered can be used to improve the intervention for implementation and nationwide roll-out. Due to its potential as an additional treatment option, this mixed methods process evaluation aims to investigate the context, implementation, and mechanisms of impact of the PFJ intervention, including feasibility, satisfaction, and usefulness of the intervention’s structure and components. Furthermore, higher adherence to the PFJ intervention’s lifestyle recommendations showed larger reductions of disease activity or symptoms compared to those with lower adherence [12, 13]. Consequently, a higher degree of implementation, as quantified by an implementation index score, is hypothesized to be associated with greater changes in healthy lifestyle behaviors [12, 13]. This study therefore also explores the association between the degree of implementation and participant’s lifestyle changes.

## Methods

A mixed methods process evaluation was conducted using the UK MRC guidelines for process evaluation of complex interventions [14] as well as previous process evaluations [15–17].

## Plants for joints study

This process evaluation is part of a larger Plants for Joints (PFJ) study conducted at the Reade rheumatology center in Amsterdam, the Netherlands. The study consisted of three randomized controlled trials which evaluated the effectiveness of the PFJ intervention on disease activity, risk score, or symptoms in people with 1) RA (*n* = 77), 2) arthralgia (at risk for RA) (*n* = 14), or 3) OA (*n* = 64), respectively, compared to usual care [18]. Participants who fulfilled the inclusion criteria were randomized to the 16-week intervention group or a usual care control group. After 16 weeks the control group received the same intervention.

The randomized controlled trials took place from May 2019 to September 2021. The Medical Ethics Review Committee of the VU University Medical Center Amsterdam (NL66649.048.18) approved the study and all participants provided written informed consent. Trial protocols were prospectively registered in the International Clinical Trial Registry Platform on 17-06-2019 with numbers NL7800, NL7801, and NL7802.

## Plants for joints intervention

The 16-week multidisciplinary lifestyle intervention consisted of theoretical and practical education on a whole-food plant-based diet, physical activity, and sleep and stress management. The PFJ intervention was delivered via 10 weekly group sessions, including a cooking workshop (for detailed information see Additional file 1), in mixed groups of participants with, or at risk for, RA, or OA, and those initially randomized to the intervention or control group.

A multidisciplinary team, each specializing in different lifestyle components, developed and interchangeably delivered the intervention, under supervision of a research dietician. Participants received an individual physical therapy consultation at the start and, optionally, end of the intervention, and, upon request coaches gave additional individual guidance. Participants received a binder with nutritional information, recipes, a meal plan, and an optional fasting protocol, a Fitbit fitness tracker, and homework exercises after each group session. Participants were encouraged to create a WhatsApp chat with their group.

Due to the COVID-19 pandemic, from March 2020 onwards the group sessions, which were initially live, were held online via Zoom. Due to varying COVID-19 restrictions some groups received a fully online or a hybrid (live and online) intervention.

## Data collection

Data for the process evaluation was collected from all participants and coaches before, during, and after the intervention. In November 2021, all participants were invited to complete an online questionnaire (Additional file 2) and participate in one of five two-hour focus groups about the intervention, conducted in December 2021 online via Zoom and led by senior qualitative researcher F.N., with A.T. and C.W.. Some participants completed the process evaluation up to two years post-intervention. Additionally, all coaches were invited to a 30 to 60-minute online interview with either F.N, A.T., or C.W. The focus groups (Additional file 3) and interviews (Additional file 4) followed a semi-structured topic guide. An overview of the research domains, data collection methods, and sources used is found in Table 1.

**Table 1:** Overview of process evaluation objectives and methods used in the Plants for Joints (PFJ) process evaluation

		**Quantitative methods**		**Qualitative methods**		
**Research objectives**		Questionnaire (participants)	Group session notes	Baseline measurement	Focus group (participants)	Interview (coaches)
	**Domain 1: Context**					
	***Reach***					
**1**	Characteristics of participants who signed up for PFJ	x		x		
**2**	Characteristics of coaches who delivered PFJ					x
	**Domain 2: Implementation**					
	***Recruitment***					
**3**	How participants heard about the PFJ intervention	x			x	
**4**	Participants' reported motivation to join PFJ, including barriers and facilitators to sign-up	x			x	
**5**	How coaches were approached to take part in PFJ					x
**6**	Coaches' motivation to join PFJ					x
**7**	Participants' experiences with the intervention before the it started, including provision of information, expectation management, starting directly or waiting to start (control group)				x	
	***Delivery***					
**8**	Number of group sessions attended by participants (dosage)		x			
**9**	Amount of intervention tools used by participants (dose received)	x				
**10**	Participants' views of the extent to which they were stimulated to use behavioral change techniques by coaches (dose-delivered)	x				
**11**	Coaches' views of the extent to which participants were stimulated to use behavioral change techniques (dose-delivered)					x
**12**	Coaches' views of the extent to which they delivered PFJ as planned/ instructed (fidelity)					x
	**Domain 3: Mechanisms of impact**					
	***Responsiveness***					
**13**	Participants' views and experiences of the tools and behavior change techniques provided during the (specific) group sessions, group setting and dynamic, feasibility, and guidance and coaching	x			x	
**14**	Coaches' views and experiences of the instructions (training) received and preparation, tools and behavior change techniques used, (specific) group sessions, group setting and dynamic, and their delivered guidance and coaching					x
**15**	Participants' overall satisfaction of the intervention	x			x	
**16**	Participants' perceived impact of the intervention on the ability to make short- and long-term lifestyle change(s)	x			x	
**17**	Participants' perceived social support and impact of the intervention on the social environment	x			x	
	**Domain 4: Facilitators and barriers**					
**18**	Coaches' perceived barriers and facilitators to implementing PFJ					x

## Context

### Reach

Participant characteristics (e.g. age, sex, body mass index (BMI), diagnosis) were assessed at baseline, while coach characteristics (e.g. age, education, work experience) were collected during the interviews.

## Implementation

### Recruitment

Participants were asked how they heard about the PFJ study (e.g. via rheumatologist, via social media) and their motivation to join (e.g. I wanted guidance with making lifestyle changes, I wanted to reduce my symptoms without more medication) in both the questionnaire and focus groups. Factors affecting their decision to sign-up (e.g. social environment, doubts) were discussed during the focus groups. During the interviews, coaches were asked how they heard about the PFJ intervention and their motivation to take part in it.

### Delivery

An overview was kept of the total number of groups, participants per group, group meeting attendance and reasons for non-attendance (e.g. work, sick, vacation), and whether the group session was online or live. In the questionnaire, participants were asked how often they were stimulated to use behavioral change techniques by the coaches using a four-point Likert scale (i.e. never, sometimes, regularly, often) and their usage of tools and activities (e.g. Fitbit, Eetmeter, recipes) on a four-point Likert scale (i.e. never, sometimes, regularly, often *or* never, 1 time, 2 times, 3 or more times). Reasons for (not) using the tools and activities were discussed during the focus groups. During the interviews, coaches were asked about the extent to which they delivered the group sessions as intended, factors affecting (perceived) fidelity, and whether delivery was adapted.

## Mechanism of impact

### Responsiveness

In the questionnaire, using four-point Likert scales, participants were asked how satisfied they were with the group meetings (i.e. very unsatisfied - very satisfied), the usefulness of the tools and activities and behavioral change techniques (i.e. very useless - very useful), and if they agreed with various statements about the content of the group meetings and group dynamic (i.e. completely disagree - completely agree). Participants were also prompted to choose the three most and least useful group meetings and to what extent the intervention helped them to eat more plant-based, eat more unprocessed, move more, be better prepared for a good night’s sleep, and relax better and more consciously (four-point Likert scale; completely disagree - completely agree), and if they were planning to continue these behaviors. Participants also indicated on a scale of 1 (most likely not) to 10 (very likely to) how likely they were to recommend the intervention to someone with the same disease and, when applicable, to what extent their partner and children made lifestyle changes (spill-over effect). Views and experiences of these elements as well as the recruitment process, social support, feasibility, and overall satisfaction of the intervention were discussed in the focus groups. In the coach interviews views and experiences of implementing the PFJ intervention were gathered including the tools and activities used, group sessions, group setting, and guidance in supporting participants to facilitate behavioral change.

## Implementation index score

A data driven implementation index score quantified the degree of implementation for participants in the PFJ intervention. Item selection was theory-driven and represented a distribution over the different process evaluation constructs: usage, quality of delivery, and responsiveness [19]. Twenty-four relevant theory-derived process-items reported by participants were included initially across the three constructs (see Table 2), i.e. 1) usage (6 items related to usage of PFJ tools and activities; never (0) – often (3)); 2) quality of delivery (12 items related to stimulus to use behavioral change techniques by coaches; never (0) – often (3)) adapted from a selection of the items used by Markland and Tobin (2010); and, 3) participant responsiveness (6 items participant’s satisfaction of the group meetings; completely disagree (0) – completely agree (3)) [20]. Greater use of PFJ tools and activities (usage), increased stimulus to use behavioral change techniques (quality of delivery), and higher satisfaction of the group meetings (participant responsiveness) were considered to contribute to a higher degree of implementation.

**Table 2:** Items included in the implementation index score

**Usage (5 items)** *Score 0 - 3* Extent to which tools and activities were used. (never, sometimes, regularly, often)	**Quality of delivery (9 items)** *Score 0 - 3* Extent to which participants felt stimulated by the coaches to use behavioral change techniques. (never, sometimes, regularly, often)	**Participant responsiveness (5 items)** *Score 0 - 3* Degree of satisfaction with group meetings. (completely disagree, disagree, agree, completely agree)
1. Food diary (Eetmeter) (7%) 2. Dietary information in binder (61%) 3. Meal plan and recipes (17%) 4. Homework (8%) 5. Whats app group (7%) **Not included:** *1. Fitbit (smart watch)*	1. Set personal goals (7%) 2. Take initiative (in your lifestyle change) (6%) 3. Ask for help if you get stuck (9%) 4. Reflect on what is going well (15%) 5. Celebrate personal victories and share these with others (12%) 6. Accept that making mistakes is part of making lifestyle changes (11%) 7. Realize that each (little) step counts (13%) 8. Trust yourself to make changes to your lifestyle (15%) 9. Come up with strategies to deal with relapses (12%) **Not included:** *1. Make lifestyle changes in a way that suits you* *2. Search for solutions and possibilities when something isn’t working* *3. Come up with strategies or make plans to deal with difficulties*	1. I did not feel very connected with the participants in my group (REV) (13%) 2. I did not really fit in with the participants in my group (REV) (46%) 3. I often felt lonely when I was with the participants in my group (REV) (11%) 4. I could talk to the participants in my group about things that are important to me (21%) 5. I had a personal connection with some of the participants in my group (10%) **Not included:** *1. I felt like I was part of the group*

Italic constructs were not considered in the composite implementation index score. Percentages in brackets refer to the proportion of each item weighing on the construct. *REV* reverse-coded item

The calculation of the implementation index score was based on previous research and analyzed with software package Mplus version 28 by conducting confirmatory factor analysis [16, 21, 22]. The validity of the three constructs (i.e. usage, quality of delivery, participant responsiveness) were tested separately, since it was not possible to add the three-factor structure in one overall construct (2nd order model) due to low power (*n* = 106). Goodness-of-fit indices (i.e. Root Mean Square Error of Approximation (RMSEA) <0.05, Standardized Root Mean Square residual (SRMR) <0.05, Comparative Fit Index (CFI) >0.95, and Chi-Square *p *>0.05) were assessed and compared. The final model had an adequate-to-good fit after deletion of items with non-significant loadings. The final PFJ implementation index consisted of 19 items divided over the three constructs with a possible index score ranging from 0 to 9 (higher score corresponds to higher degree of implementation). Each item within each construct contributed proportionally to this score and all constructs were summed to obtain an implementation index score for each participant (Table 2).

## Lifestyle changes

Lifestyle changes were quantified at weeks 0 and 16 of the intervention. Daily intake of fiber and saturated fat were used as surrogate markers to quantify adherence to a whole-food plant-based diet. Dietary intake was measured using the validated Dutch ‘Eetmeter’ by ‘Voedingscentrum’ (The Netherlands Nutrition Center) [23]. Minutes per week of physical and stress-relieving activities were collected via an online questionnaire.

## Facilitators and barriers for implementation

Coaches were asked during the interviews which factors impacted their ability to execute the intervention as intended, including facilities and collaboration with others.

## Data analysis

### Questionnaire

Participant’s characteristics and answers to the questionnaire were reported as descriptive statistics. All open-ended questions were listed and summarized.

### Focus groups and interviews

Focus group and interview audio recordings were transcribed and deductively analyzed by assigning each response to a pre-specified topic (e.g. motivation to join intervention, online vs. live group sessions) (Additional file 5). Text which was not categorized to a pre-specified topic was assigned to a new topic during analysis. Topics were sorted and grouped together into various categories and themes for a thematic analysis. Facilitators and barriers pertaining to organization and broader constructs were coded as “Other facilitators and barriers.” Analysis was performed independently by A.T. and C.W. and conflicts were resolved via discussion with F.N.

### Association between implementation and lifestyle changes

Participant characteristics were assessed in those with low, moderate, and high implementation score tertiles, and those who did not complete the process evaluation questionnaire. An explorative analysis assessed the association between implementation score and lifestyle changes using a linear regression adjusted for baseline values. Analyses were performed with R version 4.3.1 (2023-06-16) with significance set at *p* <0.05.

## Results

Of all the participants who completed the lifestyle intervention (*n* = 155), 106 (68%) completed the questionnaire and 34 (22%) took part in the focus groups (average duration 119 (range 110-129) minutes; 6-8 participants per focus group). All coaches involved in the execution of the PFJ intervention (*n* = 9) were independently interviewed (average duration 49 (range 18-66) minutes). See Supplementary Figure 1, Additional file 6 for a flow chart of the results.

## Context

### Reach

Of the 225 people assessed for eligibility, 155 completed the lifestyle intervention: RA (*n* = 77), OA (*n* = 62), at risk for RA (*n* = 14), and two people with other rheumatic conditions. The average age of process evaluation participants was 57 (SD 11; range 27 – 78) years, 87% were female, and the mean BMI was 29 (SD 6) kg/m^2^ at baseline. These baseline characteristics were similar to the whole cohort who finished the lifestyle intervention (58 (SD 12) years, 90% female, BMI 29 (SD 6) kg/m^2^).

All coaches were interviewed (*n* = 9). The group of coaches consisted of three dietitians, three sport coaches, two occupational therapists, and one physical therapist. Coaches had an average age of 41 (range 29 to 61) and seven were female. Seven coaches had prior experience giving lifestyle interventions like PFJ.

## Implementation

### Recruitment participants

Most participants heard about the PFJ study via Reade (22%), social media and/or internet (20%), and/or the newspaper or TV (25%) (multiple answers possible) (Supplementary table 1, Additional file 6). Trying to reduce my health complaints without more medication (61%), believing that lifestyle changes could influence my health complaints (57%), and prevent symptoms becoming worse (43%) were the most common reasons to sign up (multiple answers possible) (Supplementary table 2, Additional file 6).

Some participants were positively influenced from someone in their social environment or their health professional to sign up.

*“...Then a neighbor showed a piece from the newspaper and [it stated the participant] could be medication-free. Well, that appealed to me a lot. I haven’t managed that yet, but who knows. And, yes… [I signed up] because of that.” (Quote #4, Focus group (FG) 1)*

However, a few participants mentioned their rheumatologist did not support them signing up.

*“...When I told my rheumatologist, “Yeah, I’m going to do Plant for Joints: I’m going to eat vegan.” He says, “Oh, that’s all well and good, but that’s not evidence based at all.” Then I say to him, “Yeah, that’s why I’m participating, too, because it’s research.” But I kind of expected him to say, “Oh, good.” And, “Get involved.” And things like that more, but not at all. Not at all.” (#21, FG 1)*

In general, participants were satisfied with the information received before and during the intake, and stated the intake was useful, informative, and made them enthusiastic. Yet, participants felt expectation management could be improved. Specifically they wanted more information about the extent of the dietary change (emphasis on unprocessed not just vegan), the intervention’s effects on disease activity and symptoms, time required to implement the intervention and attend study measurements, and which results they would receive throughout the intervention.

### Dose delivered and received

The lifestyle intervention was given to 21 groups with an average of 7 participants per group (SD 1.6, range 5-12). Of these groups, 8 groups had all sessions live (incl. 2 groups with the last 1 or 2 sessions online) while the rest either had all sessions online (6 groups) or a hybrid form of 2-4 sessions live (including the first session) and the rest online (7 groups). Participants felt the use of behavioral change techniques were clearly integrated in the intervention. Of the behavioral change techniques used, participants were most often stimulated to take initiative of their own lifestyle changes (83%), trust themselves to make lifestyle changes (78%), and realize each little step counts (76%).

On average participants attended 8.8 (SD 1.5; range 8-10) of 10 group sessions. Most common reasons for non-attendance were work, sickness, or vacation. Tools or activities used most often were the Fitbit fitness tracker (84% used regularly or often), dietary information provided in the binder (72%), and homework exercises (84%). Participants were encouraged to use the Fitbit, with many continuing its use post-intervention, due to its insightful data and activity encouragement. While the Eetmeter provided insight, some found it time-consuming and frustrating. However, integration with personalized advice from dieticians stimulated its use. For further details on the use of behavioral change techniques and tools and activities in the PFJ intervention see Supplementary tables 3 and 4, respectively, in Additional file 6.

### Fidelity

During interviews, coaches reported delivering the intervention as intended, whereby perceived fidelity was influenced by instructions, role expectations, and participant feedback. Nutritional content and group discussions were led by the dietitians, who had a general understanding of each group session's content and were further trained by observational learning. Yet, one dietician suggested pre-established instructions would have been beneficial. Overall, a consensus existed among dieticians about the nutritional advice. Occupational therapists collaborated on relaxation and sleep session content, and felt they executed them similarly. The flexibility of the physical activity sessions allowed for adaptation based on the sport coach's preferences and perspectives, leading to improved session quality and authenticity. They collaborated about session content and evaluated them to ensure the content was cohesive. Nevertheless, one sport coach questioned the reproducibility of the sport sessions due to the individual adaptions.

*“On the other hand, because all three of us were kinda doing our own thing…I sometimes had the idea that… how reliable is it? Because it is in a research setting. If one group is training very hard with [sport coach] and the other group talks more about it or addresses it more playfully, then of course there are differences.” (#7, Sport coach)*

## Mechanism of impact

### Responsiveness

#### Group sessions

In total, 95% of participants were satisfied with the group sessions (Supplementary table 5, Additional file 6). Focus group participants expressed satisfaction with the content, amount and duration of the sessions, and ability to ask questions. In the questionnaire, only seven participants indicated dissatisfaction or disagreement with three or more questions about the usefulness of the group sessions. When asked about the specific sessions 60% of participants felt session three (information about processed foods and physical activity introduction) was most useful for making lifestyle changes followed by session one (49%; cooking workshop) and session four (38%; exercise test and relaxation exercises) (Supplementary table 6, Additional file 6). In total, 40% of the participants indicated none of the sessions were the least useful, or in other words, all were seen as useful. Coaches also felt the group sessions were an extremely useful part of the whole intervention (Supplementary table 5, Additional file 6), whereby the cooking workshop and exercise test were especially important (Supplementary table 6, Additional file 6).

### Group setting

The group setting was perceived as an essential, if not the most important part, of the lifestyle intervention, according to focus group participants. Participants and coaches felt the group sessions were fun and an opportunity to share experiences and tips, ask questions, and support and motivate each other. The coaches also noted the advice shared between participants often carried more conviction than advice provided by the coaches. The group setting also gave participants a sense of social pressure, which was generally motivational, yet some felt discouraged if they did not experience similar positive results.

*"...I don't think I could have done this whole change without doing it together with a group…we really did it with each other and stood for it together…No, I don't believe I could have done it that way individually." (#416, FG 2)*

In all focus groups, participants indicated the form in which the group sessions were held (live, online, or hybrid) impacted their satisfaction. Yet, the overall perceived usefulness was similar across all forms of the intervention. Participants with the hybrid form preferred live interaction, yet they, and those with the online form, found the online sessions a good alternative during the COVID-19 pandemic and enjoyed not having to commute. Coaches stated a hybrid form would have been ideal, allowing for live interaction while reducing travel needs, thus increasing feasibility and attendance. Informative sessions, including nutrition and sleep, as well as relaxation exercises, were viewed as suitable for online use, while live guidance of physical exercises would be preferred.

### Group dynamic

Group dynamic varied per group and depended on various factors. Overall, 86% of participants felt they were part of the group and 76% felt they could talk to their group members about things that were important to them (Table 3). In the focus groups participants who did not feel connected to their group members often indicated missing this aspect of the intervention, although not everyone felt the need for group connection.

**Table 3:** Mixed method results for the perceived group dynamic according to participants

***To what extent do you agree with the following statements about the dynamic of your group?***	Number (%) reported in participant questionnaire (*n* = 103)	**Participant experiences and quotes based on focus groups** *(n* = 34)
	**(Completely) agree**^**1**^	
I felt like I was part of the group	88 (85)	Some felt a very strong group connection which added value to their experience of the intervention. *"...The group dynamic ... I actually found the most important thing... I was [in a group] with seven participants and it was incredibly fun, but in particular because we got back together every Thursday it seemed ... just like an AA meeting, like: "Have you sinned this week?" "Well, I got a croquette from the stand…" But you did keep each other strong, and I just noticed gradually, over the weeks, that I was thinking, like: "Hey, wait a minute, I do have to stay on the right path, because otherwise I'll see them again on Thursday...Then I'll have to fess up." And I could lie about it, but that doesn't feel right, because there are all these people who are working towards the same goal. So, I was actually triggered by the group’s collective interest, or group pressure…You can see it that way. I found that very important, all the way through." (#415, FG 1)*
I could talk to the participants in my group about things that are important for me	77 (75)	Some participants shared positive experiences about being able to discuss important topics. *"I also liked working in a group, because you have a bit of support from each other. I mean, you hear stories that you yourself are also struggling with, so you don't feel like you're doing it on your own. And the positive things you share together...To have feedback and share the ups and the downs." (#246, FG 2)* On the other hand, some participants felt, for example due to different personal situations, they could not discuss important topics with their group. *"I thought: "I would have loved that, if I could have just talked to someone there." [But] in my group, there were no people in the same kind of situation." (#496, FG 2)*
I had a personal connection with some of the participants in my group	32 (31)	One participant shared their experience of individual contact with another group member and felt this added value to the intervention. *"With one person I still call from time to time. She was struggling for a while and she wanted to call every week. And that's just valuable. That you can call someone when you need support." (#419, FG 3)*
	**(Completely) disagree**^**1**^	
I did not feel very connected with the participants in my group	66 (64)	Some participants indicated they did not feel connected to others in their group or experienced a feeling of being part of a group. Of those participants some missed this aspect in the intervention. *"...that's something…which I missed a lot, the group thing."(#422, FG 1)*
I did not really fit in with the participants in my group	77 (75)	No remarks
I often felt lonely when I was with the participants in my group	96 (93)	No remarks

^1^Participants were asked to answer various statements about the group dynamic using a 4-point Linkert scale ranging from completely disagree, disagree, agree, completely agree. The sum of those reporting completely agree and agree or disagree and completely disagree are shown. *FG* focus group

One of the main factors influencing group dynamic was whether the group sessions were held live, online, or in a hybrid form. Participants who followed the live intervention felt more connected with their group (19% (live) vs. 49% (online) did not feel connected with their group) and each other (52% (live) vs. 19% (online) had a personal connection with other participants in their group) than those following the hybrid and online versions (Supplementary table 7, Additional file 6). Participants also felt it was easier to share important things in the live and hybrid forms compared to the online version. Similarly, the coaches indicated the group dynamic was better in groups which had spent more time together live.

Group diversity, including age, diagnosis, occupation, and living situation, was generally well-received by participants, and provided a wide range of perspectives and advice, which participants found valuable. However, some expressed a desire to have at least one other participant with the same diagnosis to share common experiences. Coaches noticed significant variations in knowledge, ability, and bodily complaints among participants. They felt more individualized guidance, particularly in the movement component, could improve the intervention's effectiveness. Additionally, coaches viewed gender diversity and larger group sizes (around 8 participants) as beneficial for the group dynamic.

### Tools, activities, and guidance

Of all the tools and activities offered, participants considered the Fitbit tracker and the dietary information in binder as the most useful. Comparingly, the fasting protocol, individual consultation with the physical therapist, and WhatsApp group were seen as the least useful. Further details on participants’ and coaches’ views and experiences of the tools and activities used in the intervention are described in Supplementary table 4, Additional file 6.

During the focus groups participants looked back positively on the guidance received during the intervention and felt the coaches were enthusiastic, patient, helpful, understanding, friendly, and easily accessible. Multiple participants stated the coaches’ enthusiasm, feeling heard, and being taken seriously were motivational factors.

*"...I thought everyone was nice and engaged and enthusiastic. And I also think that's kind of what makes this successful, that people are enthusiastic and genuinely interested in you and want to work with you." (#760, FG 1)*

Additionally, participants were satisfied with the ability to ask questions as well as the answers and information received by the coaches (Supplementary table 5, Additional file 6). Participants felt the guidance was pleasant without feeling pressured or pushed. A few participants also appreciated that many of the coaches ate a plant-based diet themselves, allowing the coaches to share personal experiences and practical tips. The perceived usefulness of the various behavioral change techniques used is described in Supplementary table 3, Additional file 6.

### Feasibility

The feasibility of eating more unprocessed and plant-based foods varied per individual and depended on various factors including previous diet, affinity for cooking, intake of sufficient protein and iron, costs, and social support. For those who were already eating more plant-based or cooking frequently themselves the transition was easier, although even some heavy meat-eaters indicated the transition was not difficult.

*"...I was a huge meat eater, 2.5 to 3 kilos of cheese per week, lots of eggs, lots of dairy products. I have to say, it amazed me that from day one I stopped with that and went completely vegan. It didn't take me any effort at all." (#461, FG 4)*

Participants expressed cooking plant-based, but specifically unprocessed, was more time consuming and required more creativity. One participant felt the cost of food was higher, while others indicated it was the same or even cheaper depending upon the products purchased. Also, participants stated eating at restaurants and during vacation was often more difficult.

Overall, social support strongly impacted the feasibility of the intervention for participants. Of those with a partner (74%) or children (72%), 89% of partners and 76% of children started to eat a more plant-based diet while 47% and 38% started moving more, respectively. Both participants and coaches stated involvement of partners or children made it easier to follow the intervention as participants felt supported and it was more practical.

*"...I was supported well by my wife, who joined me and made dishes with me. Yes, that has been a very big support, because I am basically a man of straw as far as that goes. So, I'm very happy that she helped me." (#714, FG 1)*

Factors affecting exercise and stress management behaviors included previous habits, symptoms and disease activity, and the COVID-19 pandemic. While COVID-19 was advantageous for some, having more time to cook and reduced travel time with online meetings, others felt staying active was harder and they were less stimulated to move due to working from home, closed gyms, and canceled group workouts. The coaches also indicated the COVID-19 pandemic increased participant’s stress, especially those with rheumatoid arthritis using immunosuppressive medication. Lastly, the coaches felt overall motivation of the participants was high, and higher than that of an average patient outside the intervention.

### Overall intervention satisfaction

Focus group participants indicated the intervention was “very good”, “fantastic”, “very complete'', and a “positive experience.” Participants found the information on lifestyle topics, including the rationale for healthy lifestyle changes, and insights given on monitoring their progress and how to listen to their bodies, the most important part.

*"...I found it very complete, that it really covers all aspects. Because it's all important, relaxation is important, exercise is important, taking small steps forward is important, nutrition is important. But social contacts are also important. So I do think [it covered] the big picture." (#303, FG 3)*

When asked how likely participants were to recommend the intervention to others with their diagnosis, they gave an average response of 9.2 (SD 1.4, *n* = 102), whereby 10 was “very likely.” Multiple participants were grateful for the intervention because it reduced their symptoms and they felt fitter. Yet, others indicated they were satisfied with the intervention even though it had no perceived impact on their symptoms.

*"I am very grateful to have been able to participate. And for me it has also been very rewarding. I would actually like everyone to be able to participate in this kind of intervention. I also really hope that rheumatologists throughout the Netherlands will be…inspired to…at least give people with rheumatism this option, to participate in such an intervention. Yes, that would be nice." (#602, FG 4)*

Coaches were satisfied with the intervention and scored it 7.5 out of 10 points (highest score). Important components of the intervention were the multidisciplinary approach, a larger reach due to the group setting, emphasis on making long-term lifestyle changes, providing insight and education, and encouraging participants to actively do and experience new things.

*"...The content is very solid. I like the variety. The holistic view of all parts of your health, not just the food, that's just really good." (#294, Dietitian)*

On the other hand, due to the scale and variety of the intervention, some participants did not have the time or capacity to address all the intervention’s components. While the intervention encouraged participants to change their lifestyle all at once, various coaches indicated it may be more useful to make smaller changes over a longer period.

Sport coaches were satisfied with the practical information given, group discussions, and lessons on protecting one’s physical boundaries. Also, movement exercises, during the sessions and as homework, were seen as one of the main stimuli for participants to be more active. Yet, sport coaches wanted more feedback from the participants to tailor sessions better. Lastly, education and group exercises were seen as important components of the relaxation and sleep components of the intervention. Yet, due to the vastness of the intervention there was little to no time to focus on behavioral change of these components.

### Perceived effectiveness

The intervention helped participants to eat a more plant-based (92%) and less processed diet (86%), move more (79%), be better equipped to ensure a good night’s sleep (66%), and relax better and more consciously (72%) (Supplementary table 8, Additional file 6). Participants' intentions to continue to follow the intervention in the future are described in Supplementary table 8, Additional file 6. Many participants found the intervention effective for improving health outcomes. Some experienced effects within a couple weeks, while for others it took multiple weeks or months, or they did not perceive any or only some effects.

## Implementation index and lifestyle changes

On average participants who completed the process evaluation questionnaire had an implementation index score of 5.7 (SD 1.3; range 3.5-8.5). Participants with RA (5.9 (1.1)) and arthralgia (6.0 (1.4)) had higher average implementation index scores than those with OA (5.4 (1.5)). Those who followed the intervention live (6.0 (1.3)) had a higher implementation index score than both those following the intervention in a hybrid form (5.5 (1.1) or online (5.6 (1.4)). Participants in the medium and high implementation score tertiles demonstrated greater changes in fiber and saturated fat intake, indicating a shift towards a more plant-based diet, as well as increased weekly physical activity, compared to the lowest implementation score tertile (Table 4). Participants who did not complete the process evaluation questionnaire had lower baseline fiber intake compared to the implementation score tertiles, while other lifestyle characteristics were similar. At the end of the intervention no significant associations were found in the linear regression analysis between the implementation index score and lifestyle changes when adjusted for baseline values (Fiber: β 0.5 (95% CI –0.7, 1.3); Saturated fat: β 0.0 (95% CI –0.0, 0.0); Physical activity: β 3.1 (95% CI –14.7, 20.9); Stress relieving activities: β 0.8 (95% CI –3.5, 5.0).

**Table 4:** Participants characteristics split up by tertiles of implementation score

		**Implementation index score (range 0 – 9)**			
	All (*n* = 155)	Low (*n* = 33)	Medium (*n* = 35)	High (*n* = 34)	Missing (*n* = 53)
Implementation index score, mean (SD)	5.7 (1.3)^a^	4.3 (0.7)	5.6 (0.3)	7.1 (0.7)	-
**Baseline characteristics**					
Age, mean (SD)	57.5 (11.5)	59.8 (10.5)	54.5 (12.5)	58.9 (11.0)	57.1 (11.6)
Female, number (%)	139 (90)	27 (82)	31 (89)	30 (88)	51 (96)
BMI (kgm^2^), mean (SD)	29.2 (6.0)	30.0 (6.1)	27.5 (5.7)	28.9 (4.8)	30.0 (6.6)
Intervention group, number (%)^b^	79 (51)	14 (42)	19 (54)	18 (53)	28 (53)
**Diagnosis, number (%)**					
Rheumatoid arthritis	77 (50)	11 (33)	21 (60)	19 (56)	26 (49)
Osteoarthritis	62 (40)	19 (58)	9 (26)	12 (35)	22 (42)
Arthralgia	14 (9)	1 (3)	5 (14)	3 (9)	5 (9)
Other	2 (1)	2 (6)	-	-	-
**Meeting type, number (%)**					
Live	50 (32)	8 (24)	9 (26)	15 (44)	18 (34)
Hybrid	54 (35)	10 (30)	16 (46)	7 (21)	21 (40)
Online	49 (32)	15 (46)	10 (39)	12 (35)	12 (23)
**Change in dietary intake, *n***	99	23	28	23	25
Fiber (g/1000kcal), median [IQR]	6.9 [3.4 – 9.6]	5.9 [3.3 – 9.1]	6.9 [4.0 – 8.3]	6.9 [2.8 –11.3]	7.4 [4.6 – 11.7]
Saturated fat (energy%), median [IQR]	–5.0 [–6.0 – (–1.0)]	–2.0 [–6.5 – (–1.5)]	–5.0 [–6.3 – (–4.0)]	–5.0 [–6.0 – (–1.0)]	–5.0 [–6.0 – (–2.0)]
**Change in physical activity, *n***	141	33	24	28	46
min/week, mean (SD)	17 (135)	–2 (136)	61 (124)	23 (107)	–6 (152)
**Change in stress relieving activities, *n***	133	29	32	28	44
min/week, mean (SD)	3 (30)	6 (23)	9 (29)	–5 (33)	3 (34)

Tertiles of the implementation index score were made (<5.09 = Low group; ≥5.09 and ≤6.1 = medium group; >6.1 = high group). Those who finished the lifestyle intervention but did not fill-in the evaluation questionnaire were classified in the missing group

^a^*n* = 102

^b^Participants who were directly randomized to receive the lifestyle intervention (did not take part in the 16-week waiting list control group before starting the intervention)

## Other facilitators and barriers

The coaches named multiple barriers impeding the execution of the intervention. First, various coaches felt the organization was “amateuristic” due to logistical problems and limited staff. Other factors such as technological difficulties and unavailable or inadequate quality of the necessary facilities or tools inhibited the execution of the group sessions. On the other hand, the collaboration between coaches was described as well organized, pleasant, and easily accessible.

*"It was very well coordinated of who was doing what. So it was very clear who was responsible for what. It was well communicated and well organized." (#195, Sports coach)*

Yet, the physical therapist had insufficient communication with the sport coaches, resulting in limited exchange of participant information and group session content, despite overlapping topics. This was seen as especially relevant as several sport coaches wished to provide more individual attention to participants.

## Discussion

This mixed methods process evaluation offers important insights into the context, implementation, and mechanisms of impact of the PFJ intervention, including its feasibility, overall high satisfaction, and (perceived) effectiveness. Working elements, including social support and self-monitoring were identified as essential to facilitate lifestyle changes. The extent to which participants implemented the intervention was assessed with the implementation index score. In an explorative analysis there was no significant difference in healthy lifestyle changes across implementation score groups.

Notably, only 9% of participants were referred to the PFJ program by their physicians. Assumptions about a patient’s lack of motivation or acceptability of dietary changes limit the use of lifestyle recommendations by health professionals in medical practice [24–26]. In this study, coaches indicated the participants taking part in the PFJ program were highly motivated whereby 57% of participants believed lifestyle changes could improve their symptoms. Despite capturing a motivated patient population in the PFJ study, in a general Dutch population over 75% of people also indicated they believe lifestyle changes can affect their health and 52% stated they want to change their lifestyle [27]. Although not everyone will be willing to make lifestyle changes, assumptions about patient’s motivation should not limit the use of lifestyle medicine in practice and rather health professionals should be encouraged to inform, motivate, and refer patients for guidance.

Social support, both in and outside of the intervention, was recognized as important to help support and motivate participants. Other group-based interventions have demonstrated that a group setting can be effective, if not more so than one-on-one guidance, at supporting various lifestyle changes and improvements in health outcomes, such as weight-loss [28, 29]. Reasons supporting the use and effectiveness of group-based interventions include positive group dynamic, social support, external motivation, and interpersonal change processes (e.g. social comparisons and validation) [28, 30]. In participant focus groups the group dynamic was seen as an important component contributing to the perceived effectiveness of the intervention. A sense of group connection led to mutual accountability, sharing of personal experiences, and increased overall satisfaction of the intervention. Yet, as PFJ participants deliberately chose to take part in a group intervention, those with a preference for individual coaching may not have chosen this intervention and their evaluation of a group-approach may differ.

Furthermore, one of the main factors affecting the group dynamic was whether the group meetings were held online or live. Participants following the intervention online felt less connected with their group and individual group members compared to participants who followed the intervention live. These findings are supported by literature showing techniques known to facilitate group dynamics, such as opportunities for informal talk and group interaction, are limited in an online setting [28]. Given the current increased popularity and feasibility of web-based lifestyle interventions, their limitations should be recognized, especially on the development of a positive group dynamic in a group-based setting.

Social support outside of the intervention (i.e. at home, with friends, at work) was also seen as an important factor affecting feasibility of the PFJ intervention. Similarly, other lifestyle interventions show superior results when involving a spouse compared to when participants were alone [31, 32]. Although partners were encouraged to attend the cooking workshop, due to COVID-19 this was not always possible. Also, some participants indicated their partner lacked practical information, thus limiting their involvement. Therefore, in the future, due to the importance of social support, spouses, and where applicable children, should be actively invited and encouraged to attend practical and theoretical components of lifestyle interventions.

Self-monitoring in the PFJ intervention, using a Fitbit fitness tracker and the Eetmeter food diary, played an important role. Both participants and coaches found the Fitbit valuable for understanding physical activity and sleep patterns, tracking progress, and incentivized moving more. Studies support these findings, showing use of a Fitbit tracker can lead to higher daily step counts, increased moderate to vigorous physical activity, and can be useful to grossly estimate quality of sleep [33, 34]. Furthermore, goal setting with the Fitbit has been found to be a promising component of the effectiveness of Fitbit-based interventions [33]. As the Fitbit was not structurally integrated within the PFJ program, its effects on behavioral change could be further optimized through structured goal setting and providing coaches with insight to facilitate more individualized feedback. Furthermore, the Eetmeter (food diary) was useful to gain insight into one’s dietary intake. This allowed participants to determine if they were meeting their recommended daily intake of various macro- and micronutrients, and enabled coaches to give personal dietary advice. Yet, some participants found the Eetmeter time-consuming and frustrating to use, limiting its accuracy and use. In the future, training participants how to fill-in a food diary, or instead using a 24-hour dietary recall or improved apps, could increase responsiveness and maximize the usefulness of dietary assessment in making behavioral change [35].

Low dietary adherence and acceptance of plant-based diets are often thought of as a concern for its use in lifestyle interventions [26]. Yet, in this study many participants felt eating a whole-food plant-based diet was easy and enjoyable, regardless of their previous diet. Moreover, the acceptability of the intervention was exemplified by a high degree of intention to continue to follow the intervention elements in the future. These findings are supported by studies who found no difference in acceptance of plant-based diets (i.e. vegan and vegetarian) when compared to diets including meat [36]. However, as the participants in the PFJ study were selected for their willingness to eat plant-based, the acceptability of the intervention may not be generalizable to the general population. Overall, though, given the acceptability and effectiveness of the PFJ intervention, and the need for a global switch towards more sustainable dietary patterns [37], implementation of this intervention within healthcare systems is overall feasible and highly relevant.

In the PFJ RCT, greater adherence to program recommendations was associated with improved disease activity or symptoms for RA and OA groups, respectively [12, 13]. Although a higher degree of implementation was hypothesized to be associated with greater lifestyle changes, our explorative analysis showed no significant difference. The non-significance could be explained by the small sample size, recall bias, or lack of variation in implementation score between participants, the later indicating good overall implementation but potentially inhibiting the ability to differentiate between participants.

### Strengths and limitations

A major strength of this study is the mixed methods approach whereby various data sources were used allowing for validation and contextualization of findings. The online questionnaire was an easy way to reach the whole cohort and allowed us to gain an overall perspective of the group and identify trends. Combining these results with those from the focus groups and interviews enhanced our understanding of the questionnaire responses. A limitation of this approach was the retrospective evaluation of the intervention, whereby some participants had completed the 16-week intervention up to two years before, potentially resulting in recall bias. On the other hand, this enabled the capture of long-term perceptions and perceived effectiveness, and to what extent participants were still following the intervention. Additionally, although the 4-point Likert scales in the questionnaire provided simplicity and ease of interpretation, reducing participant burden in the lengthy questionnaire, they also constrained participants' responses, resulting in a loss of nuance and flexibility in expressing opinions. Furthermore, the use of a theory-driven implementation index score, composed of different process evaluation constructs, was a strength allowing for an explorative analysis between degree of implementation and lifestyle changes. Yet, the implementation score needs further validation, and data for the implementation score were derived from only one questionnaire completed after implementation of the PFJ intervention, potentially leading to bias. Assessing the degree of implementation during the implementation process may have provided more insight into the degree of implementation over time. Moreover, selection bias may influence the results found in this process evaluation. While all enrolled participants were invited to take part in the process evaluation, not all chose to participate. And of those who took part in the focus groups all expressed positive views about the intervention. This could also explain why there was no association found between the implementation score index and lifestyle changes, possibly due to a small variation in the score as participants were generally motivated and enthusiastic. Lastly, an incidental finding of this process evaluation, due to the unexpected COVID-19 pandemic, was the ability to evaluate the perceived effects of hosting the group intervention online vs. live.

## Conclusions

This process evaluation, using a mixed methods design, assessed why the PFJ intervention is effective and how it can be optimized. Working elements, including the form in which the intervention was given (online or live), social support within and outside of the intervention, and self-monitoring tools were identified as important factors impacting the feasibility, satisfaction, and/or usefulness of the intervention. Participants felt empowered to take control of their own health and lifestyle outcomes and indicated the plant-based diet was feasible and intended to follow it in the future, thus supporting the use of plant-based dietary interventions for human and environmental health. In an explorative analysis there was no significant difference in healthy lifestyle changes across implementation index score groups. To guide future implementation, this process evaluation also identified improvement points, such as more individual guidance, a more personalized approach, and increased visibility of one’s progress. Overall, this process evaluation further supports the use of lifestyle interventions, like PFJ, as an additional treatment option for people RA or OA, as well as other NCDs, due to its high satisfaction, feasibility, and perceived effectiveness, in addition to its clinical effectiveness.

### Supplementary Information

**Additional file 1.** Plants for Joints Intervention. Overview of Plants for Joints intervention including content of group meetings and program timeline.

**Additional file 2. **Process evaluation questionnaire. English translation (from Dutch) of the process evaluation questionnaire for Plants for Joints participants.

**Additional file 3.** Focus group guide. Guide used for process evaluation focus groups with participants.

**Additional file 4.** Interview guide. Guide used for process evaluation interviews with coaches.

**Additional file 5.** Codebook used for thematic analysis of focus group and interview transcripts.

**Additional file 6:** **Supplementary figure 1**. Results flow chart, **Supplementary table 1**. Mixed method results for participant recruitment via questionnaire and focus groups, **Supplementary table 2**. Mixed method results for participant’s motivation to join via questionnaire and focus groups, **Supplementary table 3**. Mixed method results for stimulation and usefulness of behavioral change techniques used by coaches according to participants and coaches, **Supplementary table 4**. Mixed method results for usage and perceived usefulness of offered tools and activities according to participants and coaches, **Supplementary table 5**. Mixed method results for satisfaction of the group sessions according to participants and coaches, **Supplementary table 6**. Mixed method results for group sessions perceived as most and least useful according to participants and coaches, **Supplementary table 7**. Mixed method results for group dynamic of live, hybrid, or online group sessions according to participants, **Supplementary table 8**. Mixed method results for effect of the Plants for Joints lifestyle intervention on lifestyle changes during the intervention and in the future according to participants.

## Data Availability

The datasets used and/or analysed during the current study are available from the corresponding author (C.A.W.) on reasonable request.

## Abbreviations

PFJ: Plants for Joints
RA: Rheumatoid arthritis
OA: Osteoarthritis
BMI: Body mass index
FG: Focus group
SD: Standard deviation
IQR: Interquartile range
