# Supplementary material for: How the Plants for Joints multidisciplinary lifestyle intervention achieved its effects: a mixed methods process evaluation
Source: BMC Public Health. 2024 Apr 13;24:1034. doi: 10.1186/s12889-024-18554-2 (PMC11016213; doi:10.1186/s12889-024-18554-2)

**The Plants for Joints program**

**General**

Almost all meetings start with a group discussion with the following characteristics:

1. Central topic: nutrition
2. Group support (sharing successes and disappointments)
3. Help each other by answering questions (how do you do…?) with attention to the following topics:
   1. cooking tips
   2. exercise tips
   3. Stress management tips
   4. Motivation
   5. Social: how to deal with the environment?

**Team & roles**

- Principal investigator: supervising rheumatologist
- Research dietician: overall coordination, nutrition content, leader of group meetings
- Physical therapist: individual consultations
- Dietician: leader of group meetings and relaxation coach
- Dietetic interns
- Sleep and fatigue coaches
- ‘Movement' and exercise coaches
- Vegan chef: provided cooking workshop

**Group supervisor**

Each group gets a supervisor, this is a dietician, relaxation coach, or exercise coach. This is the fixed point of contact for the group. The groups are encouraged to help each other – even outside the program.

**Group meetings**

| **Week** | **Content - description** | **Who?** | **Time**  **(minutes)** |
| --- | --- | --- | --- |
| 0 | Individual session / intake with dietician (including various measurements);  Individual session / intake with the physical therapist;  Separate appointments are made for each. | (Research) dietician  Physical therapist | 60  30 |
| 1 | Introduction by principal investigator or research dietician;  Cooking workshop with partner/other person with supporting role.  **Online variation:** Cooking demonstration without a partner. Start with introduction and sharing goals. | Vegan chef  Dietician  Interns | 180 |
| 2 | Group discussion;  Theory nutrition (plant-based, unprocessed food, effects, incl. video for repetition at home);  Introduction stress management, relaxation exercise and mindful eating/tasting (exercise in observing without judgment) and debriefing;  Longer exercise in body awareness (emphasis on acceptance of observations/experiences during body scan) and discussion.  Homework: tests and body scan. | Dietician  Relaxation coach | 30  60  60 |
| 3 | Group chat;  Food product knowledge (including reading labels);  Sitting volleyball game (no special clothing required, breaking resistance through movement). Why exercise is important, downward spiral (including an exercise on why people have started to move less, awareness);  Exercise suggestion relaxation: variants body scan, mindful tasting, breathing space (short) and longer breathing exercise (breath as anchor and/or breathing areas ([www.reade.nl/ontspanning](http://www.reade.nl/ontspanning) or www.frodo-mindfulness.nl/audio). | Dietician  Exercise coach | 30  30  30  30 |
| 4 | Assessment of one's own possibilities, body experience with 'exercise test' (to be repeated in meeting 10, week 13);  Relaxation & Exercise, Parasympathetic and Sympathetic Nervous System (with exercise to increase awareness);  Exercises: breathing space (short moment of stopping, becoming aware and allowing) and awareness standing/walking (attention in the body low). | Dietician  Exercise coach  Relaxation coach | 60  45 |
| 5 | Group discussion: sharing successes, what is easy, what is a challenge?;  Theory of movement (Health Council of the Netherlands Exercise Guidelines 2017).  Homework (digital): integrating movement into daily life.  Exercise suggestion relaxation: variants body scan, mindful tasting, breathing space (short) and longer breathing exercise (breath as anchor and/or breathing areas ( [www.reade.nl/ontspanning](http://www.reade.nl/ontspanning) or www.frodo-mindfulness.nl/audio). | Dietician  Exercise coach | 60  60 |
| 6 | Group discussion;  Movement: reflection homework;  Sleep training: basic sleep education (including sleep hygiene) and 'Sounder Sleep' exercise. | Dietician  Relaxation coach | 60  60 |
| 7 | Group discussion: 'what do you eat on an average day or what did you eat yesterday?'  The importance of flexibility, agility, body balance, yoga and/or pilates.  Homework: advice helpful in incorporating exercises into daily life and how to deal with pitfalls during practice. Exercise suggestion: breathing space, breathing areas, choose your own favorite exercise ( [www.reade.nl/ontspanning](http://www.reade.nl/ontspanning) or www.frodo-mindfulness.nl/audio). | Dietician  Exercise coach | 60  60 |
| 8 | Potluck: participants bring or share their favorite meal;  Relaxation: breathing space, dealing with thoughts. Clear your head or allow it? Exercises: breathe as an anchor, think and feel with attention. Make an inventory of questions and wishes for the last meeting. | Dietician  Relaxation coach | 60  60 |
| 9 | Group discussion;  Basic forms of movement theory and practice/direct application (coordination, strength, speed, agility, endurance)  Homework: relaxation exercise, breathing space, browsing the stream or related, right to exist, favorite choice ([www.reade.nl/ontspanning](http://www.reade.nl/ontspanning) or www.frodo-mindfulness.nl/audio). | Dietician  Exercise coach | 60  60 |
| 13 | Group discussion (homework, preparation: what ensures that I (not) keep up with the lifestyle changes in daily practice?), celebrating successes;  Review key learning points about nutrition;  Movement: Assessment of one's own possibilities, body experience with 'movement test' (repetition of week 4);  Relaxation: breathing space. Questions and wishes, intentions/plans, how to proceed with this. Exercise is determined on the spot, in line with questions and needs of participants. | Dietician  Exercise coach  Relaxation coach | 15  30  45  60 |

**Timeline**


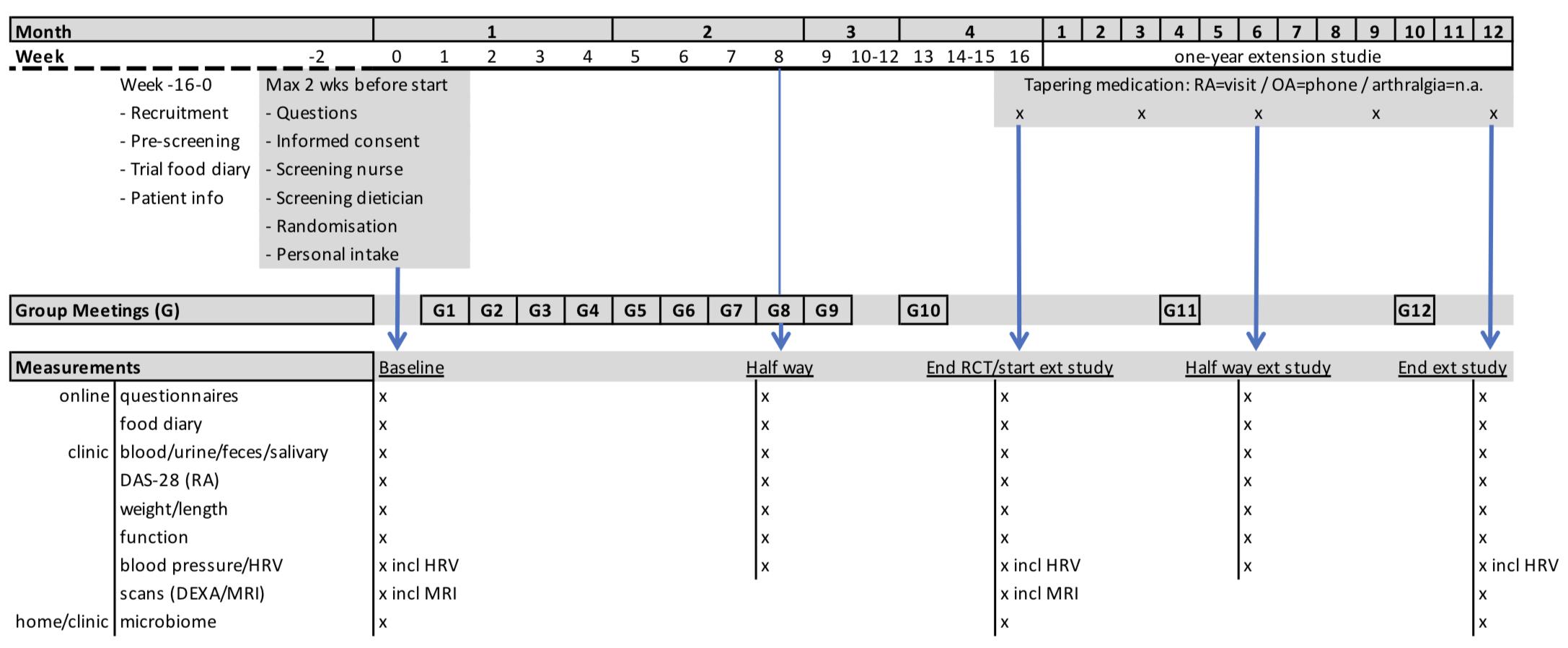

Supplement: Supplementary file 1 — Additional file 1. Plants for Joints Intervention. Overview of Plants for Joints intervention including content of group meetings and program timeline. [file 12889_2024_18554_MOESM1_ESM.docx]
