# Supplementary material for: How the Plants for Joints multidisciplinary lifestyle intervention achieved its effects: a mixed methods process evaluation
Source: BMC Public Health. 2024 Apr 13;24:1034. doi: 10.1186/s12889-024-18554-2 (PMC11016213; doi:10.1186/s12889-024-18554-2)
Supplement: Supplementary file 2 — Additional file 2. Process evaluation questionnaire. English translation (from Dutch) of the process evaluation questionnaire for Plants for Joints participants. [file 12889_2024_18554_MOESM2_ESM.pdf]

Plants for Joints - Process evaluation questionnaire

Introduction
Dear Plants for Joints participant, Thank you for completing this questionnaire about the Plants for Joints lifestyle program. It will take about 15 minutes of your time to complete this questionnaire. The questionnaire is about the activities, tools and guidance used during the Plants for Joints intervention, as well as your motivation and lifestyle changes. The answer options may be slightly different for each question, so it is important that you read each question and the answer options carefully. Important: There are no right or wrong answers, nor are there any "trick questions." We just want to know which answer you find best. Your answers will be kept confidential and used for research purposes only. Your answers are important to us!

Evaluation of the Plants for Joints program

Recruitment and motivation
1.1 How did you hear about the Plants for Joints study?
(Multiple answers possible)

- ☐ Via rheumatology center (Reade)
- ☐ Via rheumatologist
- ☐ Via social media
- ☐ The internet
- ☐ The newspaper
- ☐ Via an episode of “Doctors of Tomorrow”
- ☐ Through my social network (family, friends, acquaintances)
- ☐ Through my rehabilitation specialist, family physician, nurse practitioner, dietician, occupational therapist or physical therapist
- ☐ Other, namely:

1.2 Why did you want to take part in the Plants for Joints research?
(Multiple answers possible)

- ☐ I wanted to make lifestyle changes in a group setting
- ☐ I wanted guidance with making lifestyle changes
- ☐ I wanted to reduce my symptoms without more medication
- ☐ I wanted to prevent symptoms from progressing
- ☐ I thought lifestyle changes could influence my symptoms
- ☐ I wanted to lose weight
- ☐ I wanted to improve my general health
- ☐ I wanted to improve my blood sugar, blood pressure, and/or cholesterol
- ☐ Because of the previous positive results of Plants for Joints (from an acquaintance)
- ☐ Recommended by my doctor
- ☐ Recommended by a friend or family member
- ☐ Other, namely:

Use of tools and activities

The following questions are about the activities and resources offered to you during the first 16 weeks of the Plants for Joints lifestyle program.

How often did you use the following tools or activities?
(On EACH line, check ONE box)

	Never	Sometimes	Regularly	Often
2.1 Food (Eetmeter)	☐	☐	☐	☐
2.2 Dietary information in binder	☐	☐	☐	☐
2.3 Meal plan and recipes	☐	☐	☐	☐
2.4 Homework (exercise of medication exercises)	☐	☐	☐	☐
2.5 Fitbit fitness tracker	☐	☐	☐	☐
2.6 WhatsApp group chat	☐	☐	☐	☐

How often did you use the following tools or activities?
(On EACH line, check ONE box)

	Never	1 time	2 times	3 or more times
2.7 Individual consult with physical therapist	☐	☐	☐	☐
2.8 Individual consult with dietician	☐	☐	☐	☐
2.9 Fasting protocol	☐	☐	☐	☐

Evaluation of group sessions

How satisfied were you with the group sessions?
(On EACH line, check ONE box)

	Very unsatisfied	Unsatisfied	Satisfied	Very satisfied
3.1 The group sessions in general	☐	☐	☐	☐
3.2 The tempo in which the information was given	☐	☐	☐	☐
3.3 The ability to understand what was discussed	☐	☐	☐	☐

3.4 The length of the sessions	☐	☐	☐	☐
3.5 The number of sessions	☐	☐	☐	☐
3.6 The time between sessions	☐	☐	☐	☐
3.7 The number of participants in the group	☐	☐	☐	☐

To what extent do you agree with the following statements about the content of the group sessions?
(On EACH line, check ONE box)

	Completely disagree	Disagree	Agree	Completely agree
3.8 The topics discussed interested me	☐	☐	☐	☐
3.9 The topics were relevant to my lifestyle changes	☐	☐	☐	☐
3.10 I received enough information about the various topics	☐	☐	☐	☐
3.11 I enjoyed taking part of the group sessions	☐	☐	☐	☐
3.12 I had sufficient opportunities to ask questions	☐	☐	☐	☐
3.13 The answers and reactions to my input were useful	☐	☐	☐	☐
3.14 The information which was given by the group leaders and coaches were useful	☐	☐	☐	☐

4.1 Which group session(s) were the MOST useful for your lifestyle change?
(Please select at least one, and a maximum of three options)

- ☐ Session 1: Cooking workshop
- ☐ Session 2: How and why Plants for Joints and introduction to mindfulness
- ☐ Session 3: Information on processed foods and exercise introduction
- ☐ Session 4: Exercise test and relaxation exercises
- ☐ Session 5: Group discussion (successes and challenges) and discuss exercise recommendations
- ☐ Session 6: Group discussion (favorite products) and sleep presentation
- ☐ Session 7: Group chat (personal goals) and chair yoga
- ☐ Session 8: Potluck and relaxation
- ☐ Session 9: Summary of the important lessons discussed and discussion about types

of movement
- ☐ Session 10: Group chat (how is it going?), repetition exercise test and the future
- ☐ None of the sessions

4.2 Which group session(s) were the LEAST useful for your lifestyle change?
(Please select at least one, and a maximum of three options)

- ☐ Session 1: Cooking workshop
- ☐ Session 2: How and why Plants for Joints and introduction to mindfulness
- ☐ Session 3: Information on processed foods and exercise introduction
- ☐ Session 4: Exercise test and relaxation exercises
- ☐ Session 5: Group discussion (successes and challenges) and discuss exercise recommendations
- ☐ Session 6: Group discussion (favorite products) and sleep presentation
- ☐ Session 7: Group chat (personal goals) and chair yoga
- ☐ Session 8: Potluck and relaxation
- ☐ Session 9: Summary of the important lessons discussed and discussion about types of movement
- ☐ Session 10: Group chat (how is it going?), repetition exercise test and the future
- ☐ None of the sessions

Group dynamic
To what extent do you agree with the following statements about the dynamic of your group?
(On EACH line, check ONE box)

	Completely disagree	Disagree	Agree	Completely agree
5.1 I did not feel very connected with the participants in my group	☐	☐	☐	☐
5.2 I did not really mix with the participants in my group	☐	☐	☐	☐
5.3 I often felt lonely when I was with the participants in my group	☐	☐	☐	☐
5.4 I felt like I was part of the group	☐	☐	☐	☐
5.5 I could talk to the participants in my group about things that are important for me	☐	☐	☐	☐
5.6 I had a personal connection with some of the participants in my group	☐	☐	☐	☐

Stimulation from coaches
To what extent did you get stimulated by the group leaders and coaches to…
(On EACH line, check ONE box)

	Never	Sometimes	Regularly	Often

6.1 set personal goals	☐	☐	☐	☐
6.2 Take initiative (in your lifestyle change)	☐	☐	☐	☐
6.3 Make lifestyle changes in a way that suits you	☐	☐	☐	☐
6.4 Ask for help if you get stuck				
6.5 Search for solutions and possibilities when something isn’t working	☐	☐	☐	☐
6.6 Come up with strategies or make plans to deal with difficulties	☐	☐	☐	☐
6.7 Reflect on what is going well	☐	☐	☐	☐
6.8 Celebrate personal victories and share these with others	☐	☐	☐	☐
6.9 Accept that making mistakes is part of making lifestyle changes	☐	☐	☐	☐
6.10 Realize that each (little) step counts	☐	☐	☐	☐
6.11 Trust yourself to make changes to your lifestyle	☐	☐	☐	☐
6.12 Come up with strategies to deal with relapses	☐	☐	☐	☐

Usefulness of tools and activities

To what extent were the following tools or activities useful to make positive changes in your lifestyle?

(On EACH line, check ONE box)

	Very useless	Useless	Useful	Very useful
7.1 Intake	☐	☐	☐	☐
7.2 Group sessions	☐	☐	☐	☐
7.3 Cooking workshop	☐	☐	☐	☐
7.4 Potluck	☐	☐	☐	☐
7.5 Food (Eetmeter)	☐	☐	☐	☐

7.6 Dietary information in binder	☐	☐	☐	☐
7.7 Meal plan and recipes	☐	☐	☐	☐
7.8 Homework (exercise of medication exercises)	☐	☐	☐	☐
7.9 Supplements	☐	☐	☐	☐
7.10 WhatsApp group chat	☐	☐	☐	☐
7.11 Fitbit fitness tracker	☐	☐	☐	☐
7.12 Individual consult with dietician	☐	☐	☐	☐
7.13 Individual consult with physical therapist	☐	☐	☐	☐
7.14 Fasting protocol	☐	☐	☐	☐

To what extent were the following techniques useful to make positive changes in your lifestyle?
(On EACH line, check ONE box)

	Very useless	Useless	Useful	Very useful
8.1 Set personal goals	☐	☐	☐	☐
8.2 Take initiative (in your lifestyle change)	☐	☐	☐	☐
8.3 Make lifestyle changes in a way that suits you	☐	☐	☐	☐
8.4 Ask for help if you get stuck	☐	☐	☐	☐
8.5 Search for solutions and possibilities when something isn’t working	☐	☐	☐	☐
8.8 Come up with strategies or make plans to deal with difficulties	☐	☐	☐	☐
8.7 Reflect on what is going well	☐	☐	☐	☐
8.8 Celebrate personal victories and share these with others	☐	☐	☐	☐
8.9 Accept that making mistakes is part of making lifestyle changes	☐	☐	☐	☐

8.10 Realize that each (little) step counts	☐	☐	☐	☐
8.11 Trust yourself to make changes to your lifestyle	☐	☐	☐	☐
8.12 Come up with strategies to deal with relapses	☐	☐	☐	☐

Lifestyle changes

To what extent did your participation in the Plants for Joints lifestyle program help you with the following changes?

(On EACH line, check ONE box)

	Completely disagree	Disagree	Agree	Completely agree
9.1 To eat a more plant-based diet	☐	☐	☐	☐
9.2 To eat a less processed diet	☐	☐	☐	☐
9.3 To exercise more	☐	☐	☐	☐
9.4 To be better equipped to ensure a good night's sleep	☐	☐	☐	☐
9.5 To relax better and more consciously	☐	☐	☐	☐

Future

To what extent did your participation in the Plants for Joints lifestyle program help you with the following changes?

(On EACH line, check ONE box)

	Completely disagree	Disagree	Agree	Completely agree
9.1 I am planning to (continue to) eat a plant-based diet	☐	☐	☐	☐
9.2 I am planning to (continue to) eat an unprocessed diet	☐	☐	☐	☐
9.3 I am planning to (continue to) exercise	☐	☐	☐	☐
9.4 I am planning to (continue to) do relaxation exercises	☐	☐	☐	☐

9.5 On a scale of 1 to 10, how likely would you be to recommend this program to someone you know with your condition?

- ☐ 1 (very unlikely)
- ☐ 2
- ☐ 3
- ☐ 4
- ☐ 5
- ☐ 6
- ☐ 7
- ☐ 8
- ☐ 9
- ☐ 10 (very likely)

Involvement of social environment

The following questions are about the level of involvement of your social circle since you have participated in Plants for Joints?

11.1 If you are married or have a partner, have you noticed they have started eating more plant-based foods?

- ☐ I have no partner
- ☐ My partner has started eating more plant-based
- ☐ My partner has not started eating more plant-based

11.1.1 If you are married or have a partner, have you noticed they have started exercising more?

- ☐ I have no partner
- ☐ My partner has started moving more
- ☐ My partner has not started moving more

11.1.2 If you are married or have a partner, have you noticed they have started doing relaxation exercises more often?

- ☐ I have no partner
- ☐ My partner started doing relaxation exercises more often and/ more consciously
- ☐ My partner has not started doing relaxation exercises more often

11.2 If you have children or care for children, have you noticed they have started eating more plant-based foods?

- ☐ I do not have or care for children
- ☐ They have started eating more plant-based
- ☐ They have not started eating more plant-based

11.2.1 If you have children or care for children, have you noticed they started moving more?

- ☐ I do not have or care for children
- ☐ They have started moving more
- ☐ They have not started moving more

11.2.2 If you have children or care for children, have you noticed they started doing relaxation exercises more often?

- ☐ I do not have or care for children
- ☐ They started doing relaxation exercises more often and/ more consciously
- ☐ They have not started doing relaxation exercises more often

12.1 If you have suggestions for improving the Plants for Joints lifestyle program or comments regarding this questionnaire, please note them here:
