# Supplementary material for: How the Plants for Joints multidisciplinary lifestyle intervention achieved its effects: a mixed methods process evaluation
Source: BMC Public Health. 2024 Apr 13;24:1034. doi: 10.1186/s12889-024-18554-2 (PMC11016213; doi:10.1186/s12889-024-18554-2)
Supplement: Supplementary file 3 — Additional file 3. Focus group guide. Guide used for process evaluation focus groups with participants. [file 12889_2024_18554_MOESM3_ESM.docx]

**Plants for Joints process evaluation**

User evaluation - focus group

Set up focus groups

- 6-8 participants per group
- Duration: 2 hours, including short break 5-10 minutes
- 4 to 5 groups
- Location: online, recording via Zoom

**Topic List**

1. Motivation for participation
2. Prior information
3. Experiences lifestyle program: general, feasibility, tools and activities, guidance
4. Expectations
5. Completeness
6. Experience lifestyle change & working elements
7. Support social environment
8. Influence Corona
9. Suggestions for improvement

**Introduction and explanation**

Welcome to the focus group for Plants for Joints. This is part of an evaluation of the lifestyle program. Based on experiences, feedback and improvement suggestions, we want to improve the lifestyle program and offer it to more people in the future. The results will also be used for a publication.

*Introduce myself.*

Would you like to introduce yourself for everyone?

I will ask you in a moment if you agree to the conversation being recorded? Then we can listen back to the conversation and pick out the important things. Good to know that everything said here is kept confidential. And that statements can never be traced back to who said them.

Do you agree to the conversation being recorded? (recorder then turn on)

So we will start the focus group like this. In this conversation, I will ask all kinds of questions about Plants for Joints. About your experiences, the resources, guidance, what was helpful, what suggestions for improvement you have.

You may keep your sound on, you may also turn it off. You may respond to each other. Maybe you experienced the same thing or think very differently, we'd love to hear that. There is no right or wrong. There is really only one thing that is really important and that is that you give honest answers. We are curious about what you thought was good and what you thought was not so good. What was useful to you and what was not so useful to you.

You will find that the whole program may not be covered as we might have discussed certain components in more depth in a previous focus group..

Let's start with a round of introductions. I'll go around and ask everyone to introduce themselves as they are ordered on my screen. Then I'll ask you again if you're okay with us recording the conversation so that's on the recording as well. First we will start with a short introduction round, just so we know who is who at the moment. I can imagine that you have a lot of experiences you wish to share and we'd love to hear those as well, we'll get to that quickly in the conversation.

Would you like to introduce yourself by telling us:

- From which place in the country you are joining us?
- When you started with Plants for Joints?
- Did you attend the group meetings online or live?

X... you can start... and do you agree to us recording the conversation?

| **Domain** | **Questions** |
| --- | --- |
| *Domain: Information services* | **Information at the start**   1. How did you experience the intake interview (screening)? 2. To what extent was the information you received before starting the lifestyle program Plants for Joints sufficient? 3. Did you have sufficient time and opportunity to prepare for the lifestyle program? *Specific: difference between participants who started the intervention and participants who started in the control group.* |
| *Domain: Participation in the Plants for Joints program* | **The questions are about the first 16 weeks of the lifestyle program Plants for Joints.**  It covers the period during which the 10 group meetings were held. Various resources were also offered. The program focused on plant-based and unprocessed eating, exercise and relaxation.  **Experiences**   1. How did you experience these first 16 weeks of Plants for Joints? 2. How did you experience adjusting your lifestyle? 3. What was difficult and easy?   **Tools and activities**   1. What is your experience with the group meetings of the lifestyle program?    1. Topics    2. Attention distribution nutrition, sleep, exercise    3. Amount of information on a topic    4. Pace    5. Amount    6. Comprehensibility    7. Ability to ask questions    8. Answers and reactions to those questions 2. How did you feel about being part of a group? 3. Do you think you had enough practice or homework assignments?    1. Was it helpful?    2. Did it help you with your lifestyle change? 4. We are curious about your experiences with some of the lifestyle program tools and activities?    1. Nutrition information & recipes (folder)    2. Homework assignments    3. Fitbit    4. Physical therapist intake and follow-up    5. 1 on 1 (extra) guidance from dietician or physical therapist    6. "green" fasting   **Guidance**  During the lifestyle program you received guidance from several people: group facilitators, sleep coaches, exercise coaches, physical therapist, dieticians.   1. To what extent did you experience coherence in the program? 2. Did the topics of the sessions fit together? 3. To what extent did you experience that the guidance from the exercise coaches, physical therapist and dieticians was aligned? 4. How did you experience the guidance of the lifestyle program?    1. What went well?    2. What was less pleasant?    3. What helped you further?   **Expectations**   1. Did the lifestyle program meet your prior expectations? |
| *Domain:*  *Impact and outcome of the Plants for Joints program* | **Experience lifestyle change & working elements**   1. What aspects of the lifestyle program helped you most in your lifestyle change? *(Prompt: Group meetings, counseling, the recipe folder, etc.)* 2. What aspects from the lifestyle program helped you the least with your lifestyle change? Which part could be taken out as far as you are concerned?   Evt. If previously answered only positively:   1. Are there aspects you do not look back on positively? 2. What would you have liked to have seen done differently? 3. Were there people in your group that you know were less positive? |
| *Domain: Social support* | **Support social / environment**   1. To what extent did you receive support from your social environment during your participation in the lifestyle program? 2. What did you notice about this? (positive and/or negative). 3. Not experienced, how did you deal with it? 4. How important was this support to you? |
| *Domain:* *Impact of COVID* | **Because of corona, not all physical meetings could take place and many sessions were done online.**   1. Did COVID-19 affect your participation in Plants for Joints in other ways? |
| *Domain: Suggestions for improvement* | **Do you have ideas for improving the lifestyle program Plants for Joints?**   1. Are there aspects or topics that you have missed in the lifestyle program? 2. Are there aspects that were not in it now but that you would have liked to have been? |

*Closing remarks.* Thank participants and stop recording.
