# Supplementary material for: How the Plants for Joints multidisciplinary lifestyle intervention achieved its effects: a mixed methods process evaluation
Source: BMC Public Health. 2024 Apr 13;24:1034. doi: 10.1186/s12889-024-18554-2 (PMC11016213; doi:10.1186/s12889-024-18554-2)
Supplement: Supplementary file 4 — Additional file 4. Interview guide. Guide used for process evaluation interviews with coaches. [file 12889_2024_18554_MOESM4_ESM.docx]

**Process evaluation - interview coaches**

- Introductions, explain roles
- Explain purpose of interview
- Permission to record interview? *(start recorder)*
- Mention:
  - Your answers are confidential and anonymous
  - There is no right or wrong answer
  - Your opinion counts

| **Domain** | **Questions** |
| --- | --- |
| *Domain:  Warm up and Characteristics* | 1. Can you tell us something about yourself? 2. What is your background and education? 3. How long have you been working as a [function]? 4. How much experience do you have implementing health interventions, such as Plants for Joints? 5. What is your age? |
| *Domain: Recruitment* | 1. What was the main reason for you to start this research (only for research dietician)? 2. What was the main reason for you to take this position? 3. How did you come to this choice/decision? |
| *Domain:*  *Program delivery & fidelity* | 1. Can you explain your tasks and roles in the program? 2. To what extent are you satisfied with the instructions and/or preparation for your tasks? *(Prompt: including participants, planning measurements, performing measurements, supervising group meetings, providing content group meetings)* 3. How did you feel the implementation of the lifestyle program went? |
| *Domain: Program aspects* | 1. If you were allowed to rate the implementation/deployment of Plants for Joints, what grade between 1 and 10 would you give it? *(Prompt: What is the reason you didn't give a lower grade? Why not a 10?)* 2. How do you feel about the group meetings of the lifestyle program? *(Prompt: overall, pace, understandability, length of a meetings, time between meetings, frequency of meetings, group size & topics, amount of information with a topic, opportunity to ask questions, answers and responses to my questions).* 3. To what extent did you encourage participants to apply techniques that could be helpful in behavior change? *(Prompt: setting personal goals, taking initiative in your lifestyle change, applying changes in a way that suits you, asking for help when you got stuck, looking for solutions when something didn't work out, coming up with strategies for dealing with difficulties, dwelling on what went well, celebrating your own successes, accepting that misses and mistakes are part of lifestyle change, realizing that every (small) step matters, having confidence in yourself to adapt your lifestyle, coming up with strategies for dealing with relapse)* 4. What do you think of Plants for Joints' tools and activities? 5. If you could rate the tools and activities, what rating between 1 and 10 would you give them? *(Prompt: tracking nutrition with the Eat Meter, nutrition information (in folder), weekly menus and recipes, homework assignments at a group meeting, such as exercise and relaxation exercises, fitbit - fitness tracker, WhatsApp group chat)* 6. How do you feel about the other activities of the lifestyle program? *(Prompt: 1-on-1 supervision by a physical therapist, 1-on-1 supervision by a dietician, fasting during the program ('green fasting'))* 7. To what extent do you think the group meetings conveyed the core message to participants? 8. Which aspects in the program do you think were most helpful to the participants? Why? 9. What aspects in the program do you think were least helpful to the participants? Why? 10. How do you look back on the choice of group approach and the group dynamics and influence on it? *(Prompt: importance of group, influence of group, positive or negative group dynamics)* 11. How do you feel about the ratio of group supervision to individual supervision during the lifestyle program? 12. Do you have improvement suggestions for the lifestyle program? |

| *Domain:* *Barriers and facilitators* | 1. How was the cooperation between the different executors of the program? 2. What helped you implement your components within the program? Why? 3. What got in the way of you performing your role(s)/ tasks within the program? Why? 4. Were there any issues within Reade or within the Plants for Joints team that you felt might have affected the implementation of the lifestyle program? |
| --- | --- |
| *Domain: Sustainability* | 1. What is important for successful implementation of the lifestyle intervention? 2. What advice would you like to give to group facilitators rolling out the lifestyle program in the future? 3. Are there any other things you would like to mention? |
