# Supplementary material for: How the Plants for Joints multidisciplinary lifestyle intervention achieved its effects: a mixed methods process evaluation
Source: BMC Public Health. 2024 Apr 13;24:1034. doi: 10.1186/s12889-024-18554-2 (PMC11016213; doi:10.1186/s12889-024-18554-2)
Supplement: Supplementary file 6 — Additional file 6: Supplementary figure 1. Results flow chart, Supplementary table 1. Mixed method results for participant recruitment via questionnaire and focus groups, Supplementary table 2. Mixed method results for participant’s motivation to join via questionnaire and focus groups, Supplementary table 3. Mixed method results for stimulation and usefulness of behavioral change techniques used by coaches according to participants and coaches, Supplementary table 4. Mixed method results for usage and perceived usefulness of offered tools and activities according to participants and coaches, Supplementary table 5. Mixed method results for satisfaction of the group sessions according to participants and coaches, Supplementary table 6. Mixed method results for group sessions perceived as most and least useful according to participants and coaches, Supplementary table 7. Mixed method results for group dynamic of live, hybrid, or online group sessions according to participants, Supplementary table 8. Mixed method results for effect of the Plants for Joints lifestyle intervention on lifestyle changes during the intervention and in the future according to participants. [file 12889_2024_18554_MOESM6_ESM.pdf]

Supplementary figure and tables

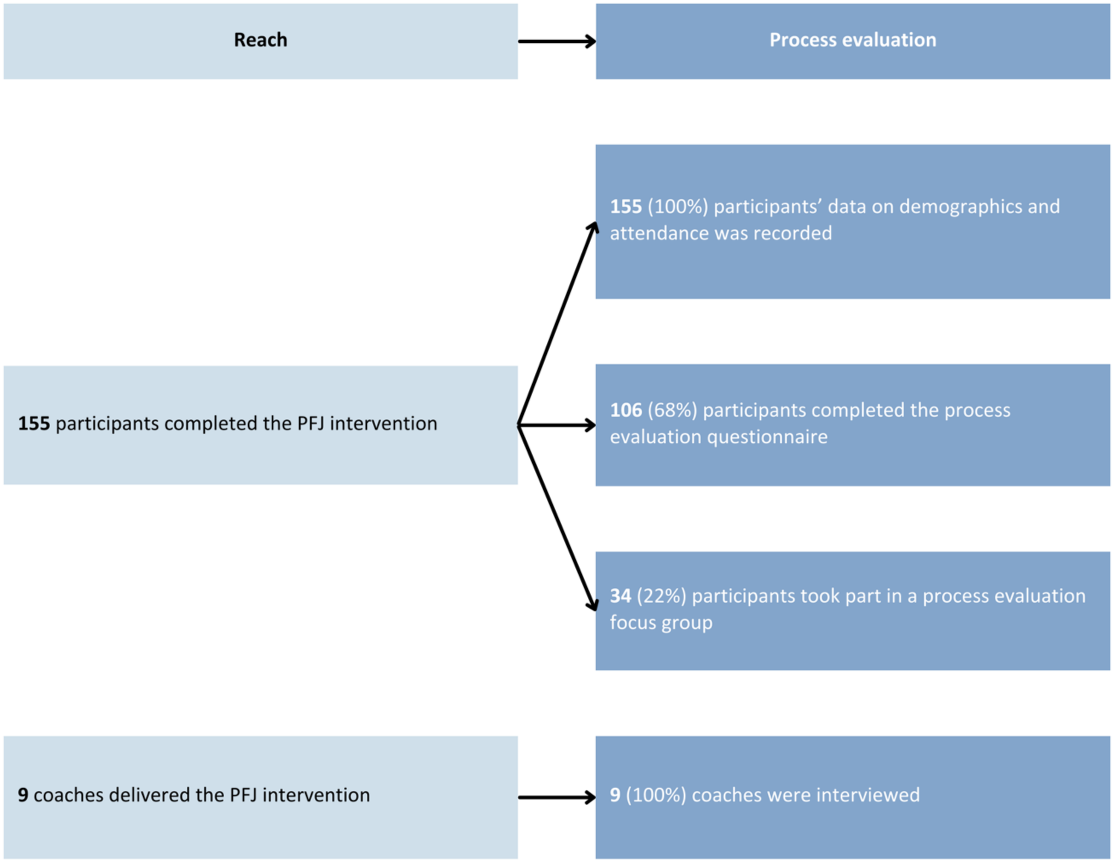

Supplementary Figure 1. Results flow chart

Supplementary table 1. Mixed method results for participant recruitment via questionnaire and focus groups.

**How did you hear about the Plants for Joints study?** *Multiple answers possible*	**Number (%) "Yes" reported in participant questionnaire** (*n* = 106)	**Participant experiences based on focus groups** (*n* = 34)	**Participant quotes**
Via rheumatology center (Reade)	23 (22)	Participants heard about the research in different ways: through a leaflet, the website or their attending physician.	*"And then there was a leaflet somewhere on the corner of his desk and in response to that I actually asked: "Is there anything I can do with food?"...And then he said: "Well, then you should see if you can do [Plants for Joints]." (#15, FG 1)*
Via rheumatologist	12 (11)	One participant mentioned it was told about Plants for Joints by its rheumatologist. In multiple focus groups some participants mentioned they missed support from their rheumatologist, inside and outside Reade, for joining the research.	*"Well, I had asked my rheumatologist many times before: "Can't I do something with nutrition?" And he said, "No, there's no scientific evidence for that." And then I also asked him to keep me informed if there would be a study on it at some point, and he didn't, even though he works at Reade. But I found out about it myself at one point, because there was a poster... Then I said: "Hey, I want to be a part of that." (#3, FG 1)*
Via the internet / social media	21 (20)	A few participants mentioned an online source for hearing about the intervention.	
Via the media (newspaper article or TV episode)	26 (25)	A few participants mentioned an article in the newspaper (Volkskrant). There were no remarks on the TV episode Dokters van Morgen.	*"And then I read in the newspaper that there was a study and with very good results. And then I thought: 'Well, I'm going to give it a try.'" (#11, FG 1)*
Other	36 (34)	For example other ways participants heard about the intervention were via family of friends and via other attending health care providers.	*"Well my general practitioner gave me the idea. They said: "Have you ever looked into eating differently?" And then I was like: "Hey, eating differently. I eat very healthy and everything." And then he said: "Well maybe it's an eye-opener." And indeed it was." (#18, FG 3)*

Supplementary table 2. Mixed method results for participant's motivation to join via questionnaire and focus groups.

**Why did you want to take part in the Plants for Joints research?** *Multiple answers possible*	**Number (%) "Yes" reported in participant questionnaire** (*n* = 106)	**Participant experiences based on focus groups** (*n* = 34)	**Participant quotes**
I wanted to make lifestyle changes in a group setting	17 (16)	No remarks	
I wanted guidance with making lifestyle changes	23 (22)	Some participants were drawn to the intervention because of the guidance that came with it.	*"...I believe that if you eat healthy…at least it helps. But what should you eat? And then what don't you eat? And then at some point you read something like:*

			"do this and not that." And yeah, then this study came along and then I thought, "Oh, yeah. This is right up my alley." (#837, FG 2)
I wanted to reduce my symptoms without more medication	65 (61)	In multiple focus groups participants explained they wanted to reduce symptoms, mainly pain and stiffness. The possibility to reduce medication was a reason to sign up for most participants. Multiple participants mentioned they had medication side-effects, and some didn't want to use medication at all.	*"...with me the biggest culprit was fatigue. That I got up in the morning with literally lead in my shoes and that I had to sleep in the afternoon when I got out of work just to get through the day. So that was one of the big reasons for me to participate." (#860, FG 2)*<br><br>*"I got in touch with Plants For Joints because I was struggling with having to take more and more medication and I didn't want to do that anymore. ..." (#835, FG 2)*<br><br>*"I started because I respond very poorly to medication for rheumatoid arthritis…this provided an opportunity for me through nutrition, to maybe do it in a different way." (#864, FG 1)*
I wanted to prevent symptoms from progressing	46 (43)	Two participants said they wanted to prevent or postpone surgery.	*" I thought, "Anything I can do to prevent [surgery], I'll do that."" (#853, FG 2)*
I thought lifestyle changes could influence my symptoms	60 (57)	In all focus groups participants mentioned they believed that or wondered if lifestyle changes could influence their disease symptoms. Some participants brought up the importance of self-direction.	*"I think I signed up for the unprocessed plant-based diet in March 2019, because I've had rheumatoid arthritis for 24 years, and thought: 'Well I have the opportunity to start doing something about my own rheumatoid arthritis'." (#836, FG 3)*
I wanted to lose weight	21 (20)	For some participants it was important to lose weight.	*"Well, I was overweight. ... So I also had to lose weight. So that was important to me, to see: "In what on earth are these calories that I'm eating?" (#205, FG 2)*
I wanted to improve my general health	33 (31)	No remarks	
I wanted to improve my blood sugar, blood pressure, or cholesterol	18 (17)	For two participants improving their cholesterol was a main reason to join in.	*"... Because I had to start using cholesterol pills and I'm so disgusted with that because I had a very bad experience with it." (#9, FG 2)*
Recommended by my doctor	10 (9)	No remarks	
Recommended by a friend or family member	3 (3)	No remarks	
Other	7 (7)	One participant also found it important to start eating plant-based because of animal suffering.	

Participants were able to choose multiple answers in the questionnaire.

Supplementary table 3. Mixed method results for stimulation and usefulness of behavioral change techniques used by coaches according to participants and coaches.

<table>
<thead>
<tr>
<th rowspan="3"></th>
<th>To what extent did you get stimulated by the group leaders and coaches to… (n = 103)</th>
<th>To what extent were the following techniques useful to make positive changes in your lifestyle? (n = 102)</th>
<th rowspan="3">Participant experiences and quotes based on focus groups (n = 34)</th>
<th rowspan="3">Group leader or coach experiences and quotes based on interviews (n = 9)</th>
</tr>
<tr>
<th colspan="2">Number (%) reported in participant questionnaire</th>
</tr>
<tr>
<th>Regularly or often[1]</th>
<th>(Very) useful[2]</th>
</tr>
</thead>
<tbody>
<tr>
<td>Set personal goals</td>
<td>76 (74)</td>
<td>98 (96)</td>
<td>One participant mentioned goal setting during the consultation with the physical therapist was especially motivating as it was a reminder to stay on track.
"...And they also asked: “how far have you come with your goals and is it all working out...What did you set out to do, did you succeed…how did you work on it?” ...I thought it was actually a good reminder. Because at that moment it had been forgotten, so I thought: Okay, I was working on that and I have to get back to it. So it was really nice that that happened." (#724, FG 3)</td>
<td rowspan="4">Coaches used the behavioral change techniques in various areas of the intervention. During the physical therapy consultation emphasis was placed on making small changes at a time, setting goals, evaluating the things which did and did not go well, motivational interviewing, and encouraging participants to ask for help. These techniques were also used in the group sessions such as setting goals and accepting that making mistakes is part of the process.
"...we set goals during session two, got acquainted and identified the reason why they were participating and set goals: “what do you hope to accomplish? So we'll come back to that at the tenth meeting”. We did that. Also…about when things get tough, that that's part of it too. Nothing has to be perfect… Between the ninth and tenth meeting we also asked, "What do you want to improve or what do you want to positively reflect on?" (#319, Dietician)

The sport coaches also stated setting goals was a big part of the movement intervention. Yet, due to the group approach it was more difficult to give personal advice and implement the behavioral change techniques.
"... Asking for help also worked, but that is one of the limitations I see in the intervention, that sometimes people find it difficult to relate a situation to themselves…Exactly on that bit I would have liked to be able to give more individual attention." (#228, Sports coach)

The movement coaches also stated they focused on both short- and long-term effects of exercise, that making lifestyle changes comes paired with setbacks, and the importance of searching for a way that works for the individual.</td>
</tr>
<tr>
<td>Take initiative (in your lifestyle change)</td>
<td>85 (83)</td>
<td>100 (98)</td>
<td>Participants took responsibility and initiative for their own lifestyle change.
"Look you have to, of course, ultimately, do it all yourself. You get everything handed to you, all the information and resources that you need. But other than that you really have to do it yourself." (#85, FG 3)</td>
</tr>
<tr>
<td>Make lifestyle changes in a way that suits you</td>
<td>77 (76)</td>
<td>99 (97)</td>
<td>Participants were given information about various topics and were able to pick and choose to what extent and how they would make changes which worked for them.
"I thought it was good to get all the information, because then you can see for yourself, “I'm doing something with this. I won't do anything with this. What feels right to me? What doesn't feel right?"" (#483, FG 5)</td>
</tr>
<tr>
<td>Ask for help if you get stuck</td>
<td>60 (59)</td>
<td>93 (91)</td>
<td>Participants were encouraged to ask for help during or outside of the group sessions when they got stuck.
"...We were almost eight weeks along and everyone was enthusiastic: one had less pain, the other could reduce their medicine, one had lost seven, eight kilos. And with me, nothing was happening. It was so demotivating, so, at some point I went to the dietician: "Yeah, what am I doing wrong?" It just turns out that my body takes much longer for it to start up, because in the end…I still lost seven pounds. Only it didn't start for me until after the eighth week that I started to notice that I was less tired and had more energy... So I'm really glad I was able to go [to the dietician] for help." (#404, FG 1)</td>
</tr>
</tbody>
</table>

Search for solutions and possibilities when something isn't working	56 (55)	93 (91)	During the group sessions participants helped each other come up with solutions to individual as well as common problems.<br>*"...the group sessions…when we checked whether we had completed our goals or not… I thought that was very good…Like: well if you didn't succeed, then maybe take it back one step. Or maybe others had ideas to help. That way we also helped each other." (#427, FG 3)*
Come up with strategies or make plans to deal with difficulties	47 (46)	90 (88)	No remarks
Reflect on what is going well	74 (73)	94 (92)	Multiple participants mentioned the use of reflecting on what is going well and the use of this to stay motivated and on track.<br>*"What I really liked about Reade, and that actually applies to almost all topics, when things don't go so well, when you think: I'm going to quit, I'm not going to do it anymore", you get dragged through it and they look at what went well, and what could maybe be a little bit better." (#769, FG 3)*
Celebrate personal victories and share these with others	61 (60)	84 (82)	During the group and outside the group sessions participants shared personal victories with their group members.<br>*"When I hear via Whatsapp that someone who had a lot of complaints stopped their medication, and is still doing it, I can really enjoy their story. That they managed to do that beautifully..." (#249, FG 2)*
Accept that making mistakes is part of making lifestyle changes	59 (58)	94 (92)	No remarks
Realize that each (little) step counts	78 (77)	98 (96)	Participants stated the use of behavioral change techniques, such as taking small steps, were clearly integrated throughout the intervention and were perceived as positive.<br>*"Those meetings where we talked about [behavioral change], I did notice a very clear line throughout all the meetings about helping and taking small steps and naming the positive. And... what you haven't reached was made a little bit less bad. And that's very nice for the people participating." (#727, FG 3)*
Trust yourself to make changes to your lifestyle	79 (78)	98 (96)	No remarks
Come up with strategies to deal with relapses	45 (44)	89 (87	No remarks

[1]Use of behavioral change techniques was assessed using a 4-point Likert scale ranging from never, sometimes, regularly, and often. The sum of those reporting regularly and often is reported. [2]Similarly, the usefulness of the techniques was assessed using a 4-point Likert scale ranging from not useful at all, not useful, useful, very useful. The sum of those reporting useful and very useful are reported. FG = focus group.

Supplementary table 4. Mixed method results for usage and perceived usefulness of offered tools and activities according to participants and coaches.

	How often did you use the following tools or activities? (*n* = 106)	***To what extent were the following tools or activities useful to make positive changes in your lifestyle?*** (*n* = 102)	**Participant experiences and quotes based on focus groups** (*n*= 34)	**Group leader or coach experiences and quotes based on interviews** (*n* = 9)
	Number (%) reported in participant questionnaire			
	**Regularly or often[1]**	**(Very) useful[2]**		
Food diary (Eetmeter)	69 (65)	85 (83)	In all focus groups the food diary (Eetmeter) was discussed. Most participants found it useful because it gave them insight into their dietary intake, especially the amount of protein, fat, and caloric intake.<br>*"I found using the Eetmeter, for example I did it for quite a long time, I just liked it, because I could take a moment to check if I was getting all the nutrients in." (#172, FG 5)*<br><br>For many participants, though, it was time-consuming because the Eetmeter asked for precise amounts. For some, meals were difficult to enter in the diary and frustrating to register because of the large number of ingredients. Also, due to the large number of brands of certain foods in the library of the Eetmeter, it was time-consuming to find the right brand, or in some cases the specific brand was missing.<br>*"And having to fill out the Eetmeter in great detail, it really frustrates me, so... I kept that to a minimum and I actually regretted that. I didn't manage to see the fun in that." (#171, FG 5)*<br><br>A few participants mentioned they received personal advice from a dietician after filling in their dietary intake in the Eetmeter which was helpful and stimulated the participants to continue to use the Eetmeter.	The food diary was used to aid participants with their lifestyle transition. Coaches reported that it also gave participants insight into their dietary habits. Using the food diary dieticians were able to see participants' progress and give feedback on their dietary intake.<br>*"...I think it's an important tool to use for people to become more aware of "how do I do that?". Also, to let participants know it is a tool which can be used in the future to check how complete their diet is. It's a nice tool for supervisors to see where the improvement points are." (#331, Dietician)*<br><br>Yet, also coaches acknowledged that the Eetmeter was seen as difficult to use for participants and needs more explanation before use.
Dietary information in binder	76 (72)	97 (95)	Overall participants felt the dietary information in the binder was useful and informative, especially at the beginning of the intervention.<br>*"...I found it very helpful. I looked in it a lot at the beginning..." (#26, FG 5)*	The information was complete but could be optimized to look more professional and up to date.<br>*"Yeah, pretty good, I guess. We could see if we could optimize it or maybe make it prettier, make it clearer, make it more practical in some ways. There are a number of products pictured, but of course that keeps*

				changing...There continue to be more meat substitutes coming out, for example." (#367, Dietician)
Meal plan and recipes	51 (48)	88 (86)	Some participants used the recipes and accompanying week menu often and felt it was very useful, especially at the start of the intervention. Others used the recipes as a guideline. Multiple participants mentioned they especially liked the breakfast options in the binder. In multiple focus groups participants mentioned they felt the recipes were a lot of work, the portions were too large, and flavors were boring, monotonous, or lacking. Participants missed more diversity of flavors and cuisines, and seasonal recipes. As a result, participants stated they often stopped following the recipes and chose meals which were simpler and with smaller portions. *"...I just found the flavor of the recipes in the book very bland. I like Asian or Mediterranean cuisines, with quite a bit of garlic or with herbs." (#227, FG 2)*	The meal plan with recipes was seen as useful as it gave participants structure and inspiration. Yet, participants did not always use it. *"I think it was very helpful. A lot of people need to know "what can I eat" or "what can I put on my bread"? …We now have a lady who asked, "what can I eat for breakfast?", while she's had the recipe book for two weeks where it's just beautifully laid out...so that's an issue, but it's been very helpful. Some have really followed the weekly/monthly menu all the way through and said: "at least then I know what I should buy and what I can eat." (#158, Dietician)*<br><br>Yet, because the recipes were very different to what participants were used to, the addition of simpler recipes might have been useful. *"Maybe the recipe book is too complicated, because they are completely new recipes with a lot of new ingredients. Many participants automatically started with what they were eating, but only left out the meat. So if you put in more simple recipes, it might help." (#26, Dietician)*
Homework (exercise or meditation exercises)	89 (84)	95 (93)	Most participants recall doing the homework exercises during the intervention period. The homework helped the participants prepare for the group sessions, for example by reflecting on what they wanted to get out of the session. *"...I always liked those preparation exercises ... Because you set yourself a goal or thought for a moment: "what do I really want?"" (#238, FG 2)*	Most, but not all, participants did the homework exercises. When completed the homework exercises were seen as a valuable addition to the intervention. Exercise coaches made videos so the participants could practice at home. These videos were seen as useful and instructive. Yet, one of the sport coaches stated they had no insight into whether the participants completed the homework exercises, which could have been improved by collecting data, such as with the Fitbit. Furthermore, by practicing exercises during the group sessions the threshold was lower to also do the exercises at home. *"[The homework exercises] were all practical exercises that we did during the group meetings, which might have lowered the threshold for them to do it at home…. It's really helpful to let them experience how something can be done, so that it's a little easier to start doing that at home." (#503, Dietician)*
WhatsApp group chat	43 (41)	65 (64)	During the intervention some groups made a WhatsApp group to exchange recipes, personal experiences, and keep in touch outside of the group sessions. Some participants had, and a couple years later still have, an active group chat, while for others the group chat died down or was never very active to begin with. Other groups never made a group chat. For some individuals the group chat was perceived as pleasant while for others it was unnecessary.	No remarks

Fitbit fitness tracker	89 (84)	92 (90)	Overall participants were enthusiastic about the Fitbit as it gave them insight into their movement, sleep, and heart rate and was a stimulus to sit less and reach a daily step goal.<br>*"The Fitbit has given me an insight into my sleep pattern. Because I always thought: I never get into deep sleep. But I get enough deep sleep at night. In that respect, I also sleep better because of that." (#281, FG 4)*<br><br>*"The Fitbit stimulated me to reach 10,000 steps." (#274, FG 3)*<br><br>Some participants continued to use the Fitbit after the intervention. Others used the Fitbit at the start of the intervention for motivation and insight and over time felt it was not necessary anymore.<br>*"I wore it every day for the first eight months, mostly to gain insight. But yeah, at some point I just knew, "Well, the days I work and I do that and I do exercises and that walk, I have about 8,000 steps. Other days ten." …in the first period I definitely found it motivating and a tool to get you going." (#272, FG 2)*<br><br>Various participants stated the Fitbit was uncomfortable and irritated the skin, especially at night and in combination with a swollen wrist due to RA. Also they felt the Fitbit was not always accurate, especially when measuring sleep, which was annoying.	The Fitbit was seen as a useful addition to the intervention. For example, it encouraged participants to make goals, such as the number of steps per day, and gave insight into one's daily movement.<br>*"It was super nice that people were getting insight into how many steps they were taking. What their heart rate was doing, what their sleep was doing, because in general these are people who don't move much and have less insight into what their body is capable of and what their body is doing. So [the Fitbit] provides support which helps say something about your fit physical condition." (#163, Sports coach)*<br><br>Yet, there was no structured use of the Fitbit within the group sessions. The sport coaches felt the Fitbit could have been used more within the movement sessions for the homework assignments and to gain insight into the movement habits of the participants.<br>*"I think we could have integrated the FitBit itself into the exercise intervention a little more. Now it was used a lot as a guide. "How many steps are you taking?" Then in a homework assignment try to take more steps than what you do on average and see how you can get that movement in. So I did use it, but it could have done so more actively..." (#163, Sports coach)*<br><br>Additionally, the physical therapist stated they often, but not consistently, used data from the Fitbit in their individual consults to gain insight into the participant's movement and to set goals.<br>*"A lot of people said they got insight from [the Fitbit]. And because so many people started talking about it…I asked how many steps they took on average, because it was nice to link back to that. But I don't know if I was very consistent with it…But I did try to include it, especially with the people who had no idea what they were doing…So it was sometimes a bit of a tool for myself as well. I said, "look at your Fitbit to see what you're doing." And also as a motivator: "you're only taking 2000 steps a day now, see if you can get that to 3000." That was also a change for the person, it's not yet the daily recommended amount, but another step closer." (#535, Physical therapist)*<br><br>Coaches stated not all participants liked the Fitbit. Specifically, participants occasionally had technical problems with synching the device, the watch band was irritating, it did not register all forms of movement accurately, and there was some uncertainty about the reliability of the sleep registration. As a result, coaches emphasized the Fitbit was just a tool and was not always accurate.<br>*"What I have to say about the Fitbit is that it's nice to get insight into your sleep pattern, but it's not the truth. But if you like it and if you want to figure it out a little bit, it's a nice tool, but if it makes you nervous or if*

				you start setting your goals based on it it has the opposite effect." (#81, Sleep coach)<br><br>One of the sleep coaches stated the Fitbit was not necessary for the sleep component of the intervention. But, if used within the intervention, the Fitbit's sleep registration requires an explanation at the start of its use to ensure participants can interpret the findings correctly.
	How often did you use the following tools or activities? (n = 106)<br><br>**Two or more times**[3]	***To what extent were the following tools or activities useful to make positive changes in your lifestyle?*** (n = 102)<br><br>**(Very) useful**		
Individual consult with dietician	32 (30)	80 (78)	Most, but not all, participants were aware about the opportunity to consult the dietitians about personal questions.<br>*"It might be a good thing to indicate: "If you get stuck, you can get 1-on-1 guidance." It might have been just me, I don't know. But it wasn't really clear to me [that extra guidance was available]." (#741, FG 3)*<br><br>In general, those who had individual guidance about personal questions felt adequately assisted and were happy the opportunity was available.	Many participants made use of the opportunity for individual guidance from the dietician. Although one of the dietitians guiding the group sessions always told the participants there was room for individual guidance, another dietician stated it may not have been emphasized often enough.<br>*"...we did mention [that individual guidance was possible], but maybe not often and clearly enough. That is possible...So we could have said that a little more clearly, because we did notice that when people knew they could use it, they quite liked to use it as well." (#127, Dietician)*
Individual consult with physical therapist	16 (15)	65 (64)	Some participants felt the consultations with the physical therapist were meaningful, eye-opening, and gave useful tips. Other participants, though, stated the consult gave them a feeling of guilt that they were not moving enough. Additionally, multiple participants were confused as they felt the consults were a separate entity which were not coupled with the rest of the intervention. The content, though, was seen as being coherent with that of the movement sessions.<br>*"I went to the physical therapist once and I still don't really know why. I didn't really get anything out of it at all. While I think that it can do a lot for you. But, everything else [in the intervention] was aligned...But, regarding the physical therapist I thought: "Why did I go there?"" (#121, FG 5)*	The physical therapist felt the individual consultations were useful for about 65% of the participants. The first session was a standard component of the intervention for everyone and focused on determining current movement patterns, movement barriers and facilitators, and goal setting. Participants were also given personalized advice. The evaluation (second) consultation was optional but was seen as useful because progress and earlier goals were evaluated and new goals were set. Some participants were hesitant to have a second consultation because they felt they had not done enough or met their goals. For some participants there was no space to implement movement into their lifestyle at the start of the intervention, while they were more ready at the second consultation.<br>*"[Second physical therapy session] was very non-binding in the beginning. People were allowed to participate; I put a little more pressure on them during the course of the project, because I was very curious as to why someone would drop out. Because it turned out that it was sometimes out of shame, that they were afraid that they did not comply or had not achieved their goals. While it is actually meant to*

				evaluate, which often revealed that they actually did much better than they had expected…or to help them get back on track with where they went wrong. Of course, it's a very large intervention, not everyone can tackle all the points right away. And sometimes people had made great strides in the area of nutrition, but had not gotten around to exercising. Well, sometimes during the second session they did get the insight that they now had room for that. So by continuing to ask, why don't you participate, I found out that sometimes they did want to participate after all." (#406, Physical therapist)<br><br>Due to the lack of communication between the physical therapist and the other coaches, specifically the movement coaches, the physical therapist felt their role was a separate entity of the intervention.<br>*"...I found my role very meaningful, but I also felt like a separate entity of the research. Which I think maybe could have been even more effective. It was also kind of unclear to a lot of people, that this was a separate part of all the sessions together, sometimes they didn't quite understand that. But then again, they are overwhelmed with all the information at the beginning. And of course I didn't always get much of what was happening in the rest of the intervention. But I did really enjoy doing it, so I'm glad how it went." (#239, Physical therapist)*
Fasting protocol	18 (17)	52 (51)	The fasting protocol was an optional part of the intervention. Experiences were varied: some did the protocol and indicated it went well, while others found it very difficult.<br>*"My experience is that I go through it. It's not a hobby that I find very easy or fun. But I do want to...keep doing it. Just purely to clean up my bowels. To just reset everything inside." (#137, FG 4)*<br><br>Some participants had not done it because they had low energy or were already losing a lot of weight. Others were planning on doing it in the future.<br>*"The fasting I find ... that's an essential part of the intervention, actually...I didn't do it because I got too skinny, but actually I should have done it and I think I'm going to do it….To do it you need that kind of extra incentive. But, no attention was actually given to that. They were already happy that we just started eating according to the intervention…" (#781, FG 5)*	Participants were told the benefits of fasting and encouraged to do so, but ultimately it was optional. There was some confusion whether participants were expected to start the intervention with the fasting protocol.<br>*"The fasting was indeed optional, we did bring it up during a group meeting. We gave them information about it and a whole sample schedule of a safe way to do it at home, given they prepare well...some were very enthusiastic about it. They said, "I want to do that right away," and it said somewhere in the documents that they should start with it before they started the plant-based eating. I couldn't find that in the information, but it was always a question…So that's a little bit of an issue in terms of communication...And some people didn't want it at all. We really didn't force them to do it. We just mentioned the benefits, the research, the evidence." (#39, Dietician)*

[1]To evaluate the frequency in which tools were used a 4-point Likert scale was used ranging from "never", "sometimes", "regularly", and "often". The sum of those reporting "regularly" and "often" are reported. [2]The extent to which the intervention tools and activities were perceived as useful was assessed via a 4-point Linkert scale ranging from "not useful at all", "not useful", "useful", and "very useful". The sum of those reporting "useful" and "very useful" are reported. [3]The frequency in which intervention components such as consultations with a dietician or the physical therapist and fasting protocol were used were assessed using a 4-point Likert scale ranging from "never", "once", "twice" and "three times or more". The sum of those reporting usage "twice or more" are reported. FG = focus group.

Supplementary table 5. Mixed method results for satisfaction of the group sessions according to participants and coaches.

How satisfied were you with the group sessions?	Number (%) reported in participant questionnaire (*n* = 106)<br>(Very) satisfied	**Participant experiences and quotes based on focus groups** (*n* = 34)	**Group leader or coach experiences and quotes based on interviews** (*n* = 9)
The group sessions in general	101 (95)	In general participants took part in the sessions with pleasure and felt the sessions were interesting, complete, and relevant.	Coaches felt the group sessions were an extremely useful part of the whole intervention. The sessions allowed for exchange of information and support from the group leaders, but also participants were actively involved by doing and experiencing new things, and sharing experiences and tips among themselves.<br>*"I really do think [an essential aspect of the intervention] was actively doing things. So for example…eating and exercising is essential and we all do it, but I noticed the relaxation component got pushed aside a little bit at first if you don't have or make time for it....During the group meetings we did practical exercises, which perhaps lowered the threshold to eventually start doing it at home as well...that has been...useful." (#503, Dietician)*
The tempo in which the information was given	101 (95)	Most participants felt the tempo in which the information was given was good, although one participant stated it was sometimes too slow.	No remarks
The ability to understand what was discussed	100 (94)	Participants felt the group sessions were easy to follow and understand.	Overall coaches felt the information was understandable for all. Multiple coaches mentioned the ability to understand information differed between group members, but these differences were adequately considered, for example by repetition of information.<br>*"Yes, of course there are people for whom it really doesn't sink in, but...the comprehensibility is good I think….Simple, not complicated, simple examples. Very much geared towards everyday life." (#262, Sleep coach)*<br><br>*"Yes, and just repeat it. Even for people who already know something, it's nice to repeat something on a regular basis, because sometimes people think they know something or understand how something is, but then if you mention it more often, it sticks better I think." (#212, Dietician)*<br><br>One of the coaches also stated they felt the participants were better able to understand the information than their patients outside of the intervention, and thus the participants' motivation and ability to comprehend was at a high level.<br>*"Well the Plants for Joints group…generally…has a little bit more capacity on average to understand than the rehab patients I see. Because I also have rehab patients where*

			understandability is a lot less… [these patients] don't end up in Plants for Joints, because they're not going to go out of their way to cook vegan food for four months. So the comprehension rate I thought was pretty high. And of course there are always people…cognitively they may understand it, but it doesn't sink in. That's still so many bridges too far." (#440, Sleep coach)
The length of the sessions	101 (95)	Multiple participants stated they felt the group sessions were too long and thus it was difficult to sit still and stay concentrated.<br>*"I thought it was too long...I just can't manage to sit that long and I also really totally...lost my focus, at the end. Then I thought: "Pooh." Whereas I actually quite liked it, but I thought it was too long." (#346, FG 5)*<br><br>One participant felt the sessions could have been more efficient as some participants were long-winded.<br>*"[Due to long-winded participants] it was also on the long side, so to speak. So it could have been more concise in that sense. Just a little more effective…but...that has to do with the type of people and maybe some people also saw it as an outing. And that's difficult, of course, but sometimes…as a discussion leader, you can respond to that as well. So yes, that might be a point of attention." (#353, FG 5)*	One of the coaches felt the two-hour sessions were adequate for both live and online variations of the intervention.<br>*"...Sometimes you think two hours is long, but face-to-face before COVID we always got there because there was always a bit more interaction, some more questions in between. And when we were going to transfer it last March to the online version due to COVID, I thought, are we going to make it in terms of time? Is it going to be too short or too long? So I thought that was quite a challenge, because you don't want to finish much too early either, but you also don't want to run too late...We never finished too early. So it always ran over a little bit. Yeah, it stays a bit trickier with an online session than live." (#504, Dietician)*<br><br>Both sleep coaches indicated the one-hour sleep session was enough for the purpose of the session: information provision. In order to get the most out of the one-hour session an instructional video was given to participants before the sleep session.<br>*"You know, an hour is short, but at the same time it's enough. It really depends entirely on the goal. You know, in that hour I was able to educate, I was able to explain things but I couldn't do interventions on behavioral change." (#573, Sleep coach)*<br><br>*"...It was always short and because of the video it got a little longer because they already got a lot of basic information in the video...And otherwise I had to cram that, along with the questions, into that one hour. So now we secretly just had two hours." (#309, Sleep coach)*
The number of sessions	103 (97)	Patients were content with the number of sessions, although some would have liked a reunion with their group after finishing the intervention.<br>*"No more or less, but maybe a little more afterwards." (#357, FG 5)*	The dieticians who led the group sessions felt the number of sessions were necessary to deliver all the information within the sessions.<br>*"I think the number of meetings… are necessary… maybe a bit less if there are more videos for example. But I also noticed, because we had put some content in videos, people don't watch too many videos in a row. So I think about ten, rather a little less, than more." (#155, Research dietician)*<br><br>One of the dieticians also remarked participants enjoyed coming together regularly and were sad when the group sessions ended. The dietitian stated they felt another session after the intervention may have been nice for the participants as a moment to evaluate how they are doing and stay motivated.<br>*"I had a feeling after that tenth meeting that they really wouldn't have minded if the group would continue to get together once a month….And not necessarily to learn new things, but just to evaluate how things are going. A bit of an incentive." (#65, Dietician)*

			One of the sport coaches stated the number of movement sessions were not enough to adequately guide participants with exercising.<br>*"If you look at the information I think the frequency was good. If you see how much people enjoyed guided exercise. Yet with the fitness tests, and when we did other things where we really got moving, I think it was too little." (#350, Sports coach)*
The time between sessions	103 (97)	One participant stated he valued the time between sessions allowing him to absorb and apply the knowledge he had learned.	One of the dieticians executing the group sessions stated they felt the participants were content with the weekly group sessions, especially at the beginning. Yet, more time between sessions, specifically near the end, would have allowed the participants to experiment more on their own and really absorb the information given in the previous session before moving onto another theme.<br>*"That people might have a little bit longer in between to think about it and implement it...Every time there is some more information coming in. So sometimes I had the idea that people were still at [the previous] group meeting in their minds while we were already further along." (#38, Dietician)*<br><br>One of the sport coaches also stated they felt the frequency of sport sessions was good.<br>*"I think that variety was very nice. It was also well coordinated in advance and I'm satisfied with that. Moving [wasn't addressed] every week, but people were sent out with an assignment to go explore this for the next two weeks and then it was alternated with sleep and with relaxation. I think that frequency is good." (#153, Sports coach)*
The number of participants in the groups	104 (98)	One participant stated they did not have a connection with her group, possibly because their group had few people.	Multiple coaches stated they preferred a group size of six to eight participants as this allows for enough individual attention. Yet, ten participants may be more financially feasible and would take into account cancellations.<br>*"Ten is fine if you just run the standard intervention and there aren't any specific questions. I think financially in the end I think it's necessary, but slightly smaller groups for proper attention. We did notice that the sessions went more smoothly if the groups were a little smaller. So that would be my preference...Then I think about six or so people." (#326, Sports coach)*
**To what extent do you agree with the following statements about the content of the group sessions?**	**(Completely) agree**		

The topics discussed interested me	102 (96)	Participants felt the topics discussed were relevant and interesting. Yet, depending on the goals of the individual, certain information was more relevant than others.<br>*"From all those sessions, but also all those assignments, you pick out things that you think are useful to you. The rest you kind of put aside. But, it's good to get the information." (#362, FG 5)*	No remarks
The topics were relevant to my lifestyle changes	99 (93)		
I received enough information about the various topics	105 (99)	In general participants were happy with the information received about the various topics.<br>*"I didn't feel that anything was missing or that anything was getting too much attention..." (#308, FG 5)*<br><br>A few participants stated they would have liked to receive more information about stress and sleep management. Also, others stated they wished more attention was paid on how to integrate relaxation into one's daily schedule.<br>*"I only got relaxation exercises and then I thought: "Yes, but how do I do that in my work? How do I do that with my supervisor, who actually does expect in terms of culture that I should always be available, that I should always be able to respond, that things are always in a hurry…Well, I would have liked a little more guidance on that…" (#504, FG 2).*<br><br>Also, some participants would have liked more repetition of information.<br>*"...It would be good to go through everything one more time. To go through another whole session like that. I think a lot more would stick and then come back." (#61, FG 3)*	Coaches were happy about the distribution of topics within the group sessions. In this way participants had a variation of activities such as group discussions but also cooking and various exercises.<br>*"...I like the fact that there are ten group meetings. So that [each meeting is] divided into pieces with two themes. So nutrition, exercise or exercise and relaxation or sleep and nutrition. So that way it's divide up nicely" (#288, Dietician)*<br><br>Yet, one of the sleep coaches felt relaxation and sleep could have gotten more attention.<br>*"...I really like the division in itself. Fifty, twenty-five, twenty-five...in my opinion if people want to get into recovery, relaxation and sleep as a basis is more important than movement, but at the same time, movement is again necessary for sleep." (#235, Sleep coach)*
I enjoyed taking part of the group sessions	100 (94)	In general participants took part in the group sessions with pleasure.<br>*"And [the group sessions] were always relaxed too... I always enjoyed going there."(#299, FG 1)*	No remarks
I had sufficient opportunities to ask questions	103 (97)	Participants felt they had enough opportunities to ask questions both during the sessions as well as before and after or by email.<br>*"Well, there was quite an emphasis on that. That if you had a question, that you could always ask it."(#315, FG 2)*<br><br>Yet, one participant felt there was more of a hurdle to ask questions online then if the sessions had been live.	Multiple coaches stated during the sessions there was enough room to ask questions and participants were encouraged to do so.<br>*"...at least for all the group meetings that I've done, I've always said: "let me know if you have any questions. There's room for individual guidance as well." So I did very much offer that myself and some did make use of it a lot or once in a while and others didn't use it at all." (#215, Dietician)*

		"It was indeed possible. True. But, maybe I'm just more of a physical contact person."(#319, FG 5)	
The answers and reactions to my input were useful	102 (96)	Participants felt like they were taken seriously when asked questions and received answers which were useful.<br>*"...You were really taken seriously and your questions were always addressed. So, I also found that a very reassuring feeling. If I'm worried about something, I get answers."(#317, FG 2)*	No remarks
The information which was given by the group leaders and coaches were useful	102 (96)	Participants felt the group leaders and coaches provided a lot of information and were knowledgeable about the topics discussed.<br>*[The dietitian] just knew a lot. Could answer all the questions. Came up with a lot of tips and alternatives and I found that very valuable." (#742, FG 4)*	No remarks

Supplementary table 6. Mixed method results for group sessions perceived as most and least useful according to participants and coaches.

**Which group session(s) was/were the most and the least useful for your lifestyle change?** *Up to 3 answers possible*	**Number (%) reported in participant questionnaire** *(n = 106)*		**Participant experiences and quotes based on focus groups** (*n* = 34)	**Group leader or coach experiences and quotes based on interviews** (*n* = 9)
	**Most useful**	**Least useful**		
Session 1: Cooking workshop	52 (49)	16 (15)	Overall participants found the cooking workshop fun, engaging, and educational. Participants stated they received useful tips and recipes and learned about new ingredients and a different way of cooking.<br>*"I've learned so much and certain things I make weekly, like the miso soup. So, that could be even more extensive as far as I'm concerned. I thought it was very good, that product information." (#660, FG 4)*<br><br>Participants also stated they would have liked to have another cooking workshop later in the intervention, allowing them to ask more in-depth questions after they have been experimenting themselves. Participants who followed the live cooking workshop stated it made them enthusiastic, was a great way to meet their group members, and allowed them to practice cooking themselves and taste how delicious plant-based food can be.<br>*"I've been cooking for… a long time. But it was just good to have that as a kickoff. Which got everybody excited, unprocessed, plant-based food doesn't have to be*	Coaches felt the cooking workshop was an important start to the intervention which inspired the participants and allowed them to experiment themselves, and discover how easy and tasty plant-based food can be.<br><br>*"That's a pretty new way of eating for a lot of people and the way of cooking. So if they experience in the beginning that it's possible, it's easy, and it's tasty. Is obviously a very important way to start." (#520, Dietician)*

			weird, but it's just delicious. I had two older guys in there saying, 'Well, it's just delicious food.'" (#371, FG 3)<br><br>Due to the COVID-19 pandemic some of the groups had a live cooking demonstration without a partner. One participant stated it was a shame their partner couldn't attend. Other groups had an online cooking demonstration whereby they were encouraged to cook along. Participants felt the tempo of the online session was often too fast and missed the group dynamic of cooking together.<br>*"We had it completely online, which I regretted. What we missed in the beginning, is obviously cooking together, because then you create a certain atmosphere where you get to know each other better." (#439, FG 2)*	
Session 2: How and why Plants for Joints and introduction to mindfulness	39 (37)	6 (6)	No remarks	No remarks
Session 3: Information about processed foods and exercise introduction	64 (60)	3 (3)	Some participants saw the transition to eating less processed foods as a challenge, even more so than eating plant-based. Yet the information given about unprocessed foods was seen as sufficient.<br>*"It's even more intense than I expected! I thought: "Yeah, you can't have sugar...as much unprocessed as possible..". But yes, I did have enough information." (#26, FG 5)*	No remarks
Session 4: Exercise test and relaxation exercises	41 (39)	11 (10)	Participants stated the exercise test gave insight into one's abilities and was thus motivational.<br>*"You had to...indicate in advance how many repetitions you think you can do of the exercise...With our group, that was a very special morning. Because, some people...underestimate themselves. So, they ended up doing all the exercises and they could do a lot more than they thought they could. But there were also people who had written down: "I can do 50 repetitions" and they only managed 30: "I also recognize that in my daily life, that I always underestimate or overestimate myself." So looking at movement in that way I thought it was very valuable. So yeah, I would definitely keep that one in there." (#380, FG 4)*<br><br>One participant stated the exercise test was too easy, but multiple stated it was too difficult and there was not enough attention to individual physical limitations. Due to the group setting participants felt pressure to perform and thus crossed their own boundaries.<br>*"And at some point there was a switch to the athletic part and it started with a test...But everybody is different, and you can't lump everybody together. And some people can do a lot. And then people keep saying: "do what you can" and so on, but if you see everyone shining around you, then you want to shine too. And then you do have pain in your wrists and so forth afterwards. Because you just cross your limits.*	Both the exercise test as well as group discussion about boundaries was perceived as useful for participants.<br>*"But what stuck with me the most, I immediately think of what helped people the most…I think the conversation and then also putting that into practice in forms of movement about learning to feel your limits and being careful with them. Because that's just something that a lot of people with rheumatoid arthritis and osteoarthritis find difficult. They are enthusiastic and they start exercising and they don't immediately notice that it might bother them for another three days. And that leads to disappointment, so the next time you think: "last time it bothered me for three days, I won't do it again." Whereas what you really want is: "Okay, this was too much but how are we going to discover what you can still do?", that whole process actually." (#387, Sports coach)*

			And I thought, there should have been, just like you do in a school class, that you can adjust the lessons, if you are good you can take it a step further and if you are less good you can take it a step further... That it should be clear from the start what you can or cannot do. There was really only one exercise that you could choose from: the difficult variation, or the less difficult variation." (#759, FG 3)<br><br>Some participants were very positive about the online fitness test while others were not motivated.<br>*"But it's still very difficult online, even with those exercises. I was standing by myself in the living room doing some knee bends. I'm thinking: 'I'm not going to do this. I'll do that at another time.'" (#406, FG 3)*<br><br>During the same session patients received a mindfulness exercise. One participant was already experienced with mindfulness exercises and thus felt it was redundant, while others enjoyed it a lot.<br>*"...I keep thinking about that group session, which I really enjoyed. We had a coach who gave some kind of yoga relaxation exercises...Then we were all given a mat and a blanket and there were two men, the rest were all ladies there. After five minutes, [those two men] were asleep...So, then you could hear them snoring. That was how we learned to relax. I really liked that (#387, FG 4).*	
Session 5: Group chat (successes and challenges) and discuss exercise recommendations	40 (38)	2 (2)	One participant felt discussing the exercise recommendations was eye opening and reassuring.<br>*"...That meeting on exercise. I also found that very enlightening. That it's not so much about: you must necessarily go to the gym three times a week. But all kinds of forms of exercise are also included. Even if it's gardening or whatever. That took the pressure off me. So, if I knew: I mowed grass and trimmed trees for 45 minutes, I didn't have to go to the gym as well. So, I could balance my life and still know that I was doing it correctly." (#475, FG 4)*	No remarks
Session 6: Group chat (favorite food products) and sleep presentation	28 (26)	15 (14)	Multiple participants felt the sleep presentation was interesting and useful.<br>*"...The most important thing I learned there...is that you have to...downshift during the day as well. So,you shouldn't be very busy all day and then expect at night that you can just go to bed and sleep, but during the day, for example when you eat lunch, don't read the newspaper, but look out the window...That was very helpful for me." (#391, FG 1)*<br><br>Yet, for some participants it was unremarkable, or the guidance was insufficient.<br>*"I only remember that there was a lady who gave that information and she didn't know us or make any attempt to get to know us. She did her lesson and that was it. So [the information] went in one ear and out the other." (#763, FG3)*	Overall, the sleep coaches were content with the sleep session. As they only had one hour they provided information about sleep and answered questions, but there was little to no room for further guidance within the session.<br>*"It's really focused on education and asking questions and kind of understanding what and how you can affect sleep or how you can promote sleep, but other than that it's not an intervention, because you can't do that in one hour." (#30, Sleep coach)*<br><br>One of the sleep coaches remarked that the interest in the sleep session varied between participants.

Session 7: Group chat (personal goals) and chair yoga	22 (21)	12 (11)	One participant mentioned the group chat with goal setting as well the group dynamic was motivational to make lifestyle changes. *"...the sessions…when we checked whether you achieved your goal, or did not achieve them, I thought that was very good. And noticed that the line continued. Like: if you didn't succeed, then maybe take one step back. Or: maybe you all have other suggestions? That way we also helped each other. So in those meetings with group discussions, I noticed that helping and taking small steps and naming the positive was very clear throughout all the meetings. And, the things that you haven't achieved, that was actually made a little bit less bad. And that's very nice for the people who participate." (#427, FG 3)*	The movement sessions, such as the chair yoga session, inspired participants to try different forms of movement to discover ones which were suitable for them. *"...iWe had a chair yoga session on the theme of yoga, which we then turned into chair yoga, so that everyone could join in. People were inspired by that and discovered that chair yoga was an appropriate form of exercise for them. So that was a very concrete example of giving people ideas." (Sports coach)*
Session 8: Potluck and relaxation	11 (10)	14 (13)	One participant stated they felt it was difficult to relax in a group setting.	No remarks
Session 9: Summary of the important lessons discussed and discussion about types of movement	23 (22)	10 (9)	No remarks	No remarks
Session 10: Group chat (how is it going?), repetition exercise test and the future	18 (17)	7 (7)	No remarks	No remarks
None of the sessions	0 (0)	42 (40)	No remarks	No remarks

Participants were asked to choose minimal one and up to three of their perceived most or least useful group sessions in the questionnaire.

Supplementary table 7. Mixed method results for group dynamic of live, hybrid, or online group sessions according to participants.

	Type of group sessions				Participant experiences and quotes based on focus groups (*n* = 34)
	**Total** (*n*=103)	**Live** (*n*=31)	**Hybrid** (*n*=34)	**Online** (*n*=37)	
**To what extent do you agree with the following statements about the dynamic of your group?**	**Number (%) reported (Completely) agree**[1]				
I did not feel very connected with the participants in my group	37 (36)	6 (19)	12 (35)	18 (49)	Multiple participants who followed the online or hybrid intervention felt they missed the opportunity to get to know their group members due to the virtual group sessions. *"Online I sometimes found a bit unfortunate, because I think in those group sessions, those few times we got together, you got a little more contact with your group as well, whereas online that remains a little distant. There was no other way, due to COVID, but in the future if it would be a bit more on location, it would be more fun as far as I'm concerned..." (#338, FG 5)*
I did not really mix with the participants in my group	26 (25)	5 (16)	10 (29)	11 (30)	No remarks
I often felt lonely when I was with the participants in my group	7 (7)	2 (7)	2 (6)	3 (8)	No remarks
I felt like I was part of the group	88 (85)	28 (90)	28 (82)	31 (84)	Some participants who had followed the intervention live indicated seeing each other during and outside of the group sessions was of added value as it facilitated a strong group feeling, connectedness, and the exchange of personal experiences. *"...we physically got together as a group from the beginning. Actually everyone was almost always there. Eventually we also created a group app where we are still in touch with each other. So all the meetings where we have to get blood drawn and have a check, we try to arrange it so that we are all in Amsterdam on the same day...I think it has added value. Then you have a kind of bond with each other. Because, we know quite a lot about each other. If there's something, it will be texted or told." (#426, FG 4)*
I could talk to the participants in my group about things that are important for me	77 (75)	24 (77)	27 (79)	25 (68)	Some participants preferred the live group sessions because they could share more with their group members than they would if they had been online. *"I attended the meetings physically. I found that very positive. I didn't like having it online. Because, you share more with each other than when you are online." (#329, FG 4)*
I had a personal connection with some of the participants in my group	32 (31)	16 (52)	8 (24)	7 (19)	Both participants of hybrid and online interventions stated it was a lot more difficult to connect to others on a personal level due to the virtual setting. *"We didn't cook together in the beginning, but we had a cooking workshop, where it was demonstrated. And we did two movement sessions, live at a distance. But I do agree: you're just much less likely to have one-on-ones, because [online] we only had central conversations and not just one-on-ones. So that group feeling…wasn't there." (#342, FG 2)*

[1]Participants were asked to answer various statements about the group dynamic using a 4-point Linkert scale ranging from completely disagree, disagree, agree, completely agree. The sum of those reporting completely agree and agree are shown.

Supplementary table 8. Mixed method results for effect of the Plants for Joints lifestyle intervention on lifestyle changes during the intervention and in the future according to participants.

**To what extent did your participation in the Plants for Joints lifestyle intervention help you with the following changes?**	**Number (%) reported in participant questionnaire** (*n* = 102)<br><br>**(Completely) agree**[1]	**Participant experiences and quotes based on focus groups** (*n* = 34)
To eat a more plant-based diet	93 (91)	The participants indicated the intervention taught them how to choose and prepare healthier options, think more about food and its impact on health, and introduced them to new products and a different way of eating.<br>*"You are introduced to other products. Because you never actually looked at anything to see what it contained. And that taught you…you were actually sometimes shocked by products that you thought: "that contains a lot of sugar or salt."...And before you didn't look at it at all, but now you pay more attention. You do get made aware of that, to look more closely and then choose certain kinds of food better." (#464, FG 1)*
To eat a less processed diet	88 (86)	
To exercise more	81 (79)	The intervention was effective at stimulating participants to move more because it gave insight into one's abilities and limitations, showed them different forms of movement, gave them structure on how to integrate movement into their daily schedule, and taught them why moving was essential regardless of diagnosis.<br>*"I was actually moving less and less, because I started listening to my body: if you have pain, you're going to do less and less. And the intervention convinced me that you should still move. And I thought that was really good, that I was told that so clearly, so I just started moving a lot more again." (#470, FG 1)*
To be better equipped to ensure a good night's sleep	67 (66)	Participants indicated the relaxation and sleep sessions taught them the importance of integrating relaxation moments into one's day, gave them useful tips, and gave them awareness about their own stress and sleep habits.<br>*"...I received an awful lot of tools and they also set me on a further quest. Among which is indeed relaxing. Because first you have to become aware that you have stress, even if you think you don't and then what can you do about it and how do you notice it. And I find that a fun challenge to be working on." (#67, FG 3)*<br><br>Due to the intervention some participants indicated they are able to sleep and relax better, while for others it is still difficult and practicing mindfulness did not become a daily habit.<br>*"It also dilutes very quickly. The meditating, the sleeping, et cetera. So, …that comes and goes. If I think again: "I sleep badly." Then I like to meditate. But, it hasn't become a regular pattern for me." (#487, FG 5)*
To relax better and more consciously	73 (72)	

<table>
<tr><th>To what extent do you agree with the following statements about the future?</th><th>(Completely) agree[1]</th><th></th></tr>
<tr><td>I am planning to (continue to) eat a plant-based diet</td><td>89 (87)</td><td rowspan="2">For some the focus groups took place up to 3 years after the 16-week intervention. Many of the participants indicated they were still eating (almost entirely) whole-food plant-based and intended to continue to do so.
"And then I started Plants for Joints. And that's now - I think - three years back. I'm still vegan and actually have been for two to, two-and-a-half, three years symptom-free. And medication-free." (#539, FG 1)

For some participants, re-integration of animal and processed foods caused an increase in symptoms, thus stimulating them to continue eating a whole-food plant-based diet.
"Because you see and feel results, it's also very easy to stick with it. And I've been trying it out on the weekends, one time I ate with my friend, and I found out that pork and beef, it just made me incredibly tired. I had a nice weekend, but then the rest of the week I was dragging my feet. And back then you'd eat it once a day and then in the morning my sandwich spread would be ham or smoked beef or whatever. But now that I don't do that anymore, I immediately notice a difference again, like: "Okay, I'm back to my old level." And that's very stimulating. Then you know what you're doing it for." (#538, FG 1)

Over time though, other participants indicated they became less obsessive about following a strict whole-food plant-based diet by choosing simpler recipes or including some processed vegan or animal products into their diet.
"I still eat totally plant-based and I'm going to continue to do that. I'm never going to eat an animal again, but I've gotten a little easier with the occasional burger and the occasional fake cheese… in the beginning the whole "unprocessed", that really everything, that you had to make all that yourself, that was indeed quite a lot of work. But it has also become more normal, I now find it easy. It's become a habit and everyone has joined in and it has become normal." (#544, FG 5)

Yet, one participant indicated that this resulted in them eating even more plant-based as it was more feasible.
"'Well, if I have to eat this all my life, I don't know if I'm going to keep it up.'" And then I thought, "Well, I'm going to do something less strict. Hey, I'm going to eat more of the foods that I'm used to and then see in the Eetmeter if that's going to match up a little bit with what I need to eat." And I liked that. I actually eat…more plant-based now than I did then." (#221, FG 2)

A few participants also stated other factors such as the environmental and ethical reasons to eat more plant-based were motivating to keep eating this way.
"And for me it's also for the animals, also for the environment. That has come into play a lot more for me. That's why I keep eating the way I'm eating now." (#611, FG 5)</td></tr>
<tr><td>I am planning to (continue to) eat an unprocessed diet</td><td>92 (90)</td></tr>
<tr><td>I am planning to (continue to) exercise</td><td>100 (98)</td><td>Some participants indicated they were still trying to reach their daily step-goal, practice various forms of exercise regularly, or do the movement exercises from the intervention.
"But in terms of exercise, I still do every morning, all these years, I do twenty exercises from the intervention we did. So I maintain that on a daily basis now. And yeah, exercise that's just the most important thing for me, from what I was doing then besides the plant-based foods." (#537, FG 2)</td></tr>
<tr><td>I am planning to (continue to) do relaxation exercises</td><td>84 (82)</td><td>Finding moments to practice mindfulness or rest was still part of some participant's daily schedule.
"I relax when I can, usually when I get home from work. Then I try to lie on the couch for at least half an hour, stretched out on my back. Either I fall asleep or I stay relaxed, but I make sure my body is resting. And if I don't do that, then I'm completely wrecked the next day. So, I keep it up." (#545, FG 1)

Yet, for others they really had to remind themselves to take a break and often forgot or struggled to do so.
"I keep it all up. Eating is no problem at all. Nor is moving. But, also because of my job, the less sitting, and stress,...and not always being "on", I find one of the hardest things to do." (#558, FG 2)</td></tr>
</table>

[1]Participants were asked to answer various statements about the effect of the Plants for Joints lifestyle intervention on lifestyle changes during the intervention and in intentions for the future using a 4-point Linkert scale ranging from completely disagree, disagree, agree, completely agree. The sum of those reporting completely agree and agree are shown.
